# Programmable Deuteration of Indoles via Reverse Deuterium Exchange

**DOI:** 10.1021/acs.joc.3c00819

**Published:** 2023-07-21

**Authors:** Liam S. Fitzgerald, Rachael L. McNulty, Andrew Greener, Miriam L. O’Duill

**Affiliations:** † School of Chemistry, University of Nottingham, University Park, Nottingham NG7 2RD, U.K..; ‡ School of Chemistry, University of Galway, University Road, Galway H91 TK33, Ireland

## Abstract

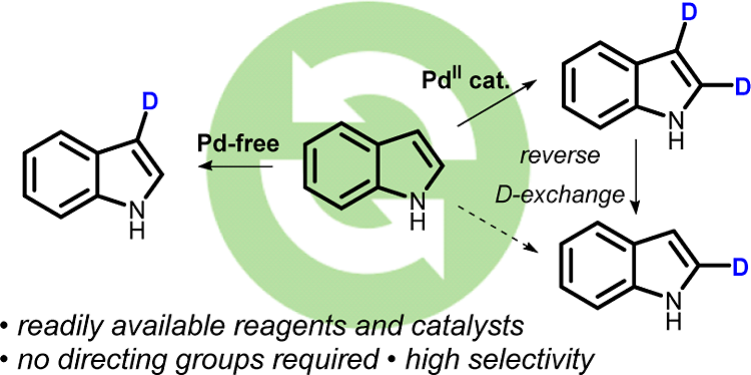

Methods for selective deuterium incorporation into drug-like molecules have become extremely valuable due to the commercial, mechanistic, and biological importance of deuterated compounds. Herein, we report a programmable labeling platform that allows access to C2, C3, or C2- and C3-deuterated indoles under mild, user-friendly conditions. The C2-deuterated indoles are accessed using a reverse hydrogen isotope exchange strategy which represents the first non-directed C2-deuteration of indoles.

Deuteration is a crucial tool for drug absorption, distribution, metabolism, and excretion studies and biomolecular analysis techniques.^1^ It can improve the metabolic stability, pharmacokinetics, and toxicity profile of drugs.^2,3^ The landmark ruling by the U.S. Food and Drug Administration classifying deuterated drugs as new chemical entities has also added significant commercial importance to these compounds.^4,5^ The development of new methods for selective deuterium incorporation into drug molecules has become an increasingly vital tool for drug discovery. A substrate class of particular interest in this area are nitrogen heterocycles—including indoles—due to their importance in many small-molecule drugs.^6−8^

Given the importance of deuterated indoles, a significant amount of work has gone into developing strategies for their deuteration (Scheme 1A).^9^ Heterogeneous catalysis generally affords perdeuterated products.^10−14^ Deuteration at C2 and C3 has been achieved with ruthenium nanoparticles,^15^ rhodium,^16^ or under homogeneous Ag_2_CO_3_ catalysis with chiral phosphine ligands.^17^ Under acid/base-mediated conditions, selective deuterium-incorporation at C3 is controlled by the molecule’s intrinsic reactivity (**2**).^18−24^ Selective functionalization of indoles at C2 (**3**) is inherently difficult and can currently only be achieved when the C3 position is blocked or by using directed approaches, i.e., *ortho*-lithiation^18^ or transition-metal-catalyzed methods requiring a directing group on the indole nitrogen.^25−30^ Directing group removal is not always straightforward^31^ and can cause isotopic dilution.^29,32^ Therefore, a directing group free method for C2-deuteration would be highly beneficial.^33,34^ Herein, we report a programmable approach allowing access to C2 (**5**), C3 (**2**), or C2-and-C3 (**4**)-deuterated indoles under mild conditions, taking advantage of innate indole reactivity and Pd(OAc)_2_ catalysis without the need for directing groups (Scheme 1B).

**Scheme 1:**
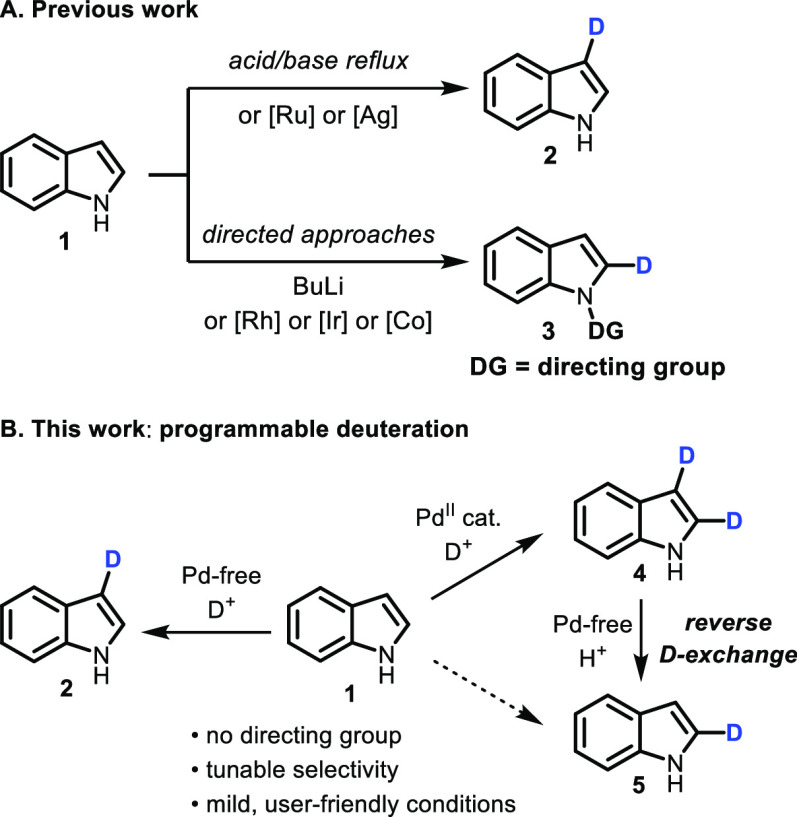
Regioselective Indole Deuteration

## Results and Discussion: Reaction Development

**Scheme 2:**
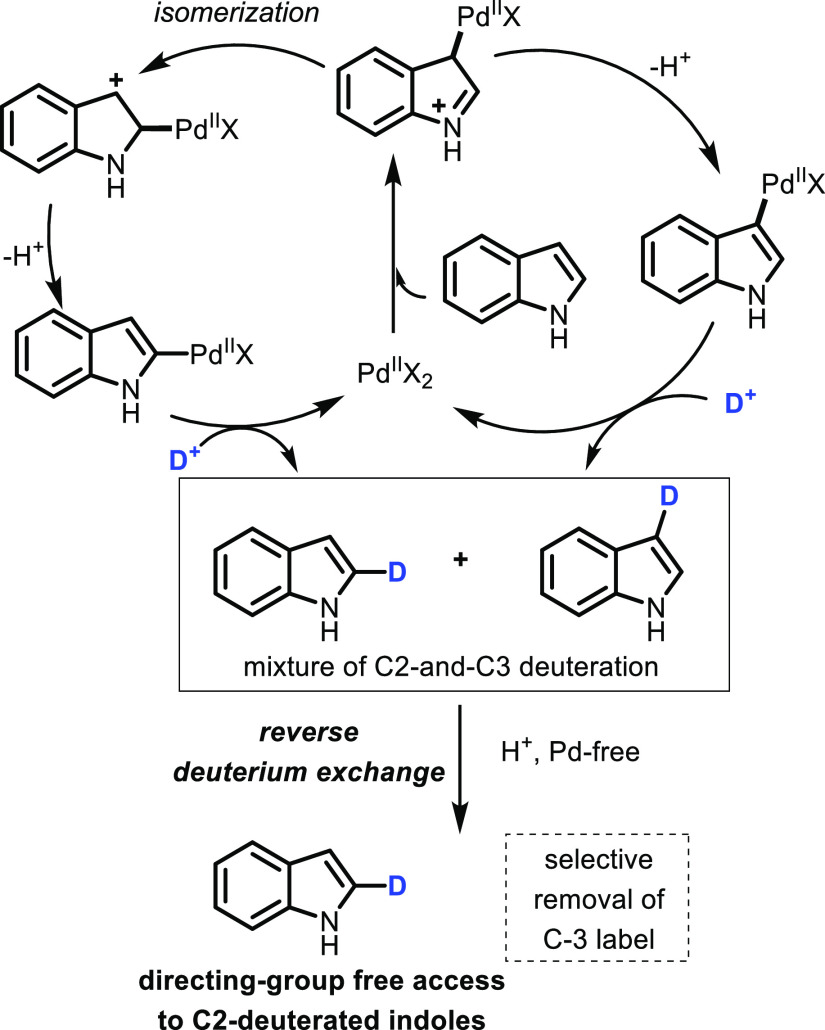
Proposed Strategy for C2-Selective Deuteration by Reverse Deuterium Exchange

Based on previous work by Gaunt,^35^ Sames,^18^ and others,^1,36−38^ we hypothesized that palladium-catalyzed deuteration would afford a mixture of C2- and C3-deuterated products through the pathways depicted in Scheme 2. However, we also reasoned that we could exploit the innate reactivity of indole in the absence of palladium to affect reverse deuterium exchange at C3.^39^ Such an approach was attractive as it provides a simple route to C2-deuterated indoles without recourse to protecting groups.

We were delighted to find that this was indeed the case. When indole reacted in the presence of 10 mol % Pd(OAc)_2_ in an anhydrous dioxane/CD_3_CO_2_D solvent mixture at 120 °C for 16 h, we observed 40% C2-deuteration and 52% C3-deuteration by ^1^H NMR spectroscopy, with no hydrogen isotope exchange at any other position (Table 1, entry 1). Deuterium incorporation increased at both positions in the presence of NaOAc to 81% (C2) and 72% (C3), respectively (entry 2). Reducing the amount of deuterated acetic acid (entries 3–4) only had a small effect on C2-deuteration, but a significant reduction of deuterium incorporation at the C3 position is observed, suggesting that the Pd-catalyzed process becomes more important as acid-mediated deuteration at C3 is slowed. Lower reaction temperatures, alternative deuterium sources, different solvents, and a reduced reaction time all had a negative effect on the reaction outcome (see the Supporting Information).

**Table 1:**
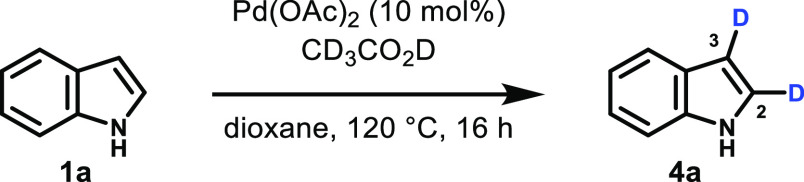
Reaction Optimization^a^

	CD_3_CO_2_D (mL)	additive	C2-deuteration [%]	C3-deuteration [%]
1	1.14		40	52
2	1.14	NaOAc (1.5 equiv)	81	72
3	0.6	NaOAc (1.5 equiv)	80	70
4	0.3	NaOAc (1.5 equiv)	70	25
5	0.6	NaOAc (15 equiv)	80	0
6^b^	0.6	NaOAc (1.5 equiv), then: K_2_CO_3_ (1 equiv), MeOH/H_2_O	81	0
7^c^	0.6	NaOAc (1.5 equiv)	0	50

a Reaction conditions: **1a** (0.2 mmol), Pd(OAc)_2_ (10 mol %), CD_3_CO_2_D, 1,4-dioxane (1.5 mL), NaOAc (1.5 equiv), 120 °C, 16 h. Deuterium incorporation determined by ^1^H NMR (see the Supporting Information).

b Reaction conditions: (i) **1a** (0.2 mmol), Pd(OAc)_2_ (10 mol %), CD_3_CO_2_D (0.6 mL), 1,4-dioxane (1.5 mL), NaOAc (1.5 equiv), 120 °C, 16 h; (ii) K_2_CO_3_ (1 equiv.), MeOH/H_2_O (0.5 mL / 1 mL), 100 °C, 16 h.

c No Pd catalyst.

Next, we attempted to achieve reverse hydrogen isotope exchange at C3 to access the C2-deuterated indole. Careful balancing of pH in one pot required a large excess of NaOAc (15 equiv), which afforded complete protonation at C3 while deuterium incorporation at C2 remained high (80%, entry 5). Due to the impracticalities of using a large excess of base on scale, we wanted to reduce this amount. Indeed, further optimization revealed that a similar result could be obtained with a telescoped approach using milder conditions: indole was reacted with 10 mol % Pd(OAc)_2_ in anhydrous CD_3_CO_2_D/dioxane at 120 °C for 16 h. After filtration through silica and removal of solvents in vacuo, 1 equiv. K_2_CO_3_ and protic solvents (MeOH/H_2_O) were added and reacted for a further 16 h (entry 6), resulting in 81% deuterium incorporation at C2 (with no isotopic labeling at C3).

A control reaction confirmed that the palladium catalyst was necessary for C2-deuteration: as expected, in the absence of Pd(OAc)_2_, only C3-deuteration was observed (entry 7).

## Reaction Scope

**Scheme 3:**
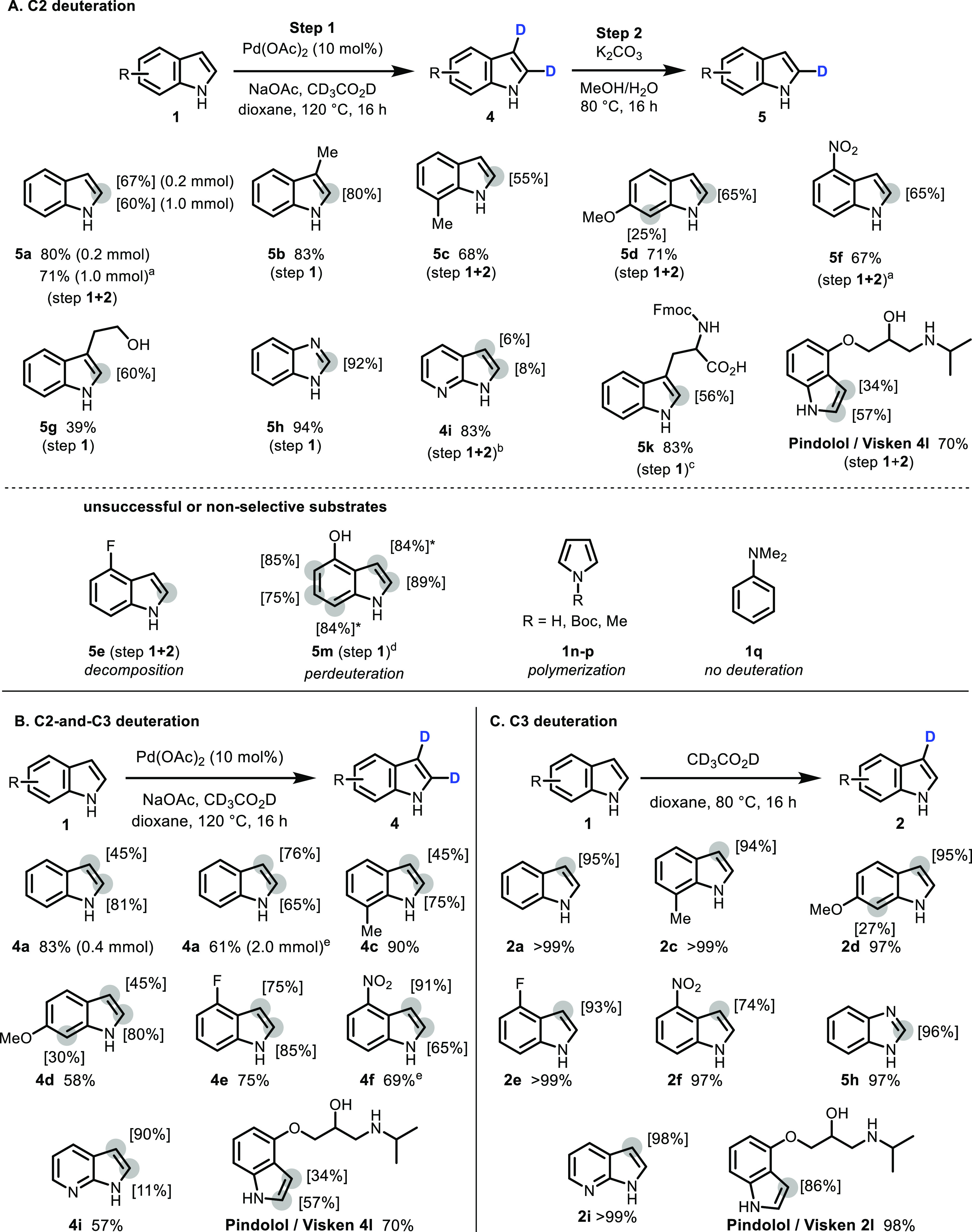
Selective C2 (A), C3 (C), and C2- and C3- (B) Deuteration of Indoles Gray circles show the labeling positions, with values in square brackets denoting isotope incorporation, as determined by ^1^H NMR. Yields and deuteration values are given for isolated products. (A) C2-deuteration conditions: Step 1: **1** (0.4 mmol), Pd(OAc)_2_ (10 mol %), NaOAc (0.6 mmol), CD_3_CO_2_D/dioxane (1.2 mL/3 mL), 120 °C, 16 h. Step 2: K_2_CO_3_ (0.2 mmol), MeOH/H_2_O (1.2 mL / 0.4 mL), 80 °C, 16 h. [a] Step 1: reaction time = 32 h. [b] Step 2 was carried out twice. [c] From Fmoc-Trp(Boc)-OH. Reaction proceeded with deprotection of the *N*-Boc group. [d] ^1^H NMR signals for H_3_ and H_7_ overlap, and an average value is given for deuterium incorporation across both positions. (B) C2- and C3-deuteration conditions: **1** (0.4 mmol), Pd(OAc)_2_ (10 mol %), NaOAc (0.6 mmol), CD_3_CO_2_D (1.2 mL), 1,4-dioxane (3 mL), 120 °C, 16 h. Higher C3-deuteration levels were observed before purification by silica flash column chromatography. [e] Reaction time = 32 h. C. C3-deuteration conditions: **1** (0.4 mmol), CD_3_CO_2_D (1.2 mL), 1,4-dioxane (3 mL), 80 °C, 16 h.

With optimized conditions in hand, we explored the substrate scope of indoles amenable to this selective C2-deuteration methodology (Scheme 3).

Indoles with no substituent on the pyrrole ring were submitted to palladium-catalyzed deuteration (method A, step 1), followed by reaction with K_2_CO_3_ in MeOH/H_2_O after purification (step 2), while indoles with a substituent on C3 did not require this second step (Scheme 3A).

Medium to high deuterium incorporation at C2 was achieved for unsubstituted indole (**5a**), as well as indoles bearing electron-donating methyl and methoxy substituents (**5b–5d**). When **5a** was scaled up from 0.2 to 1.0 mmol, longer reaction times were required in step 1 to sustain deuterium incorporation levels. Some unselective background deuteration was observed for more electron-rich indoles: 6-methoxyindole (**1d**) underwent 25% C7-deuteration, while 4-hydroxyindole showed unselective deuteration on all free positions (**5m**). Similarly, the methodology could not be extended to highly electron-rich pyrrole **1n–p** (with or without protecting groups on nitrogen) which polymerized under the reaction conditions, or other electron-rich aromatics such as *N*,*N*-dimethylaniline **1q**, which was recovered without any deuteration. For electron-poor 4-nitroindole, lower levels of deuterium incorporation were observed (**5f**: 32% deuteration at C2), but this result could be improved (to 65%) by increasing the reaction time of step 1 to 32 h. To the best of our knowledge, there are no other examples of selective C2-deuteration of electron-poor 4-nitroindole in the literature. Initial screening results for 4-fluoroindole looked promising (79% deuterium incorporation), but the compound decomposed in our hands after treatment with K_2_CO_3_. Free hydroxyl groups were tolerated, albeit with lower yields (**5g**: 39%). Free NH groups are not tolerated and require protection (see **5k** below). Benzimidazole showed high deuterium incorporation at C2 under these conditions (**5h**: 92%). However, this result is comparable to results obtained under palladium-free conditions (see Scheme 3C), suggesting that simple acid-mediated H/D exchange may be taking place. For 7-azaindole, C2 incorporation was low, possibly due to protonation or the coordination of the palladium catalyst to the nitrogen heterocycle: **4i** was formed with only 8% deuterium incorporation at C2 and 47% at C3, with C3-deuteration reduced to 6% when submitted to K_2_CO_3_ twice. To further demonstrate the utility of our methodology for late-stage functionalization, we submitted two high-value products to our deuteration conditions. The Boc/Fmoc-protected amino acid tryptophan showed 56% deuteration at C2 (**5k**) with concomitant Boc deprotection, a promising result for the use of this methodology on indole alkaloid natural products. We believe that this is the first example of C2-selective tryptophan deuteration. Efficient C2- and C3-deuteration of the clinically approved beta blocker pindolol/Visken^40^ was achieved (**4l**: 57% D incorporation at C2 and 34% at C3); however, no reduction of C3-deuteration was observed after treatment with K_2_CO_3_, even when 3 equivalents of the base were used.

Many of the C2- and C3-deuterated intermediates **4** have not previously been reported in the literature. If these compounds are desired, they can easily be isolated after the palladium-catalyzed deuteration step (Scheme 3B). C3-deuteration under these conditions was very high (75–95%) for all substrates; however, purification by silica flash column chromatography was required which reduced the deuterium content at C3 to the values shown in Scheme 3B (e.g., from 70 to 45% for **4a**). This effect was less pronounced for electron-poor indoles **4e**, **4f**, and **4i**. Deuteration of pindolol was achieved in 57 and 34% at C2 and C3, respectively. The use of anhydrous dioxane or the addition of molecular sieves was of the utmost importance to avoid isotopic dilution at C3 (see Supporting Information, Table S5). Interestingly, some isotopic dilution also seemed to occur at C2 in the reverse deuterium exchange reaction of certain electron-rich indoles (e.g., **4a**/**5a**, **4b**/**5b**, **4d**/**5d**, Scheme 3A,B). Further studies are required to explain this observation.

As seen in Table 1, no C2-deuteration occurs in the absence of palladium. Selectively C3-deuterated indoles can thus be obtained in quantitative yields by treatment with CD_3_CO_2_D under palladium-free conditions (Scheme 3C). The products from this palladium-free method did not require purification by column chromatography, which resulted in high C3-deuteration values (74–98%) across the board.

With only small modifications to the reaction conditions, selective access to C2, C3, or C2- and C3-deuterated indoles has been achieved, providing a user-friendly, programmable scaffold for selective late-stage deuteration.

## Conclusions

We have described a regiodivergent methodology for the selective deuteration of indoles. Directing-group-free palladium catalysis in the presence of deuterated acetic acid allows for hydrogen isotope exchange at the C2 and C3 position with high levels of deuterium incorporation. Reaction of these compounds with K_2_CO_3_ and a protic solvent selectively removes the isotopic label at C3, yielding C2-deuterated products. Metal-free, acid-mediated deuteration instead affords selective isotope incorporation at C3 only. The methodology allows for selective, regiodivergent late-stage deuteration of drug targets (as demonstrated on pindolol/Visken). On a more fundamental level, our reverse hydrogen isotope exchange strategy enables selective deuteration of bioactive molecules with automatic removal of labels in positions that are likely to be labile in vivo.

## Data Availability

The data underlying this study are available in the published article and its Supporting Information.

## Supporting Information Available

Experimental procedures and analytical data of products (PDF)
