# Supplementary material for: Programmable Deuteration of Indoles via Reverse Deuterium Exchange
Source: J Org Chem. 2023 Jul 21;88(15):10772–6. doi: 10.1021/acs.joc.3c00819 (PMC10407927; doi:10.1021/acs.joc.3c00819)

SUPPORTING INFORMATION

Programmable deuteration of indoles via reverse deuterium exchange

Liam S. Fitzgerald,[b] Rachael L. McNulty,[b] Andrew Greener,[a] Miriam L. O'Duill[a]*

a School of Chemistry, University of Nottingham, University Park, Nottingham NG7 2RD, UK.

b School of Chemistry, University of Galway, University Road, Galway, H91 TK33, Ireland.

* *Corresponding author: miriam.oduill@nottingham.ac.uk*

TABLE OF CONTENTS

General Information S2

Reaction Information S3

Reaction Optimisation S3

The Importance of Dry Solvents S5

Optimized Reaction Conditions S6

Method A: C2-Deuteration. S6

Method B: C3-Deuteration. S6

Characterization of Compounds S7

C2-Deuterated Indoles S7

C2-and-C3-Deuterated Indoles S10

C3-Deuterated Indoles S12

NMR Spectra S15

C2-Deuterated Indoles S15

C2- and C3-Deuterated Indoles S25

C3-Deuterated Indoles S34

General Information

All solvents and chemicals were used as purchased unless stated otherwise; all solvents were dried according to conventional methods. Anhydrous 1,4-dioxane was purchased from Sigma Aldrich and further dried over molecular sieves. NaOAc was dried at room temperature under vacuum for 4 hours prior to use. CD_3CO_2D was purchased from Deutero. All reactions were performed in oven dried apparatus with magnetic stirring under an inert atmosphere of argon or nitrogen. The reactions were followed by thin layer chromatography (TLC) carried out on aluminium-foil backed plates coated with silica gel (Merck Kieselgel 60 F_{254}). The products were visualized using UV fluorescence (254 nm) or potassium permanganate stain. Silica flash column chromatography was performed over Merck silica gel C60 (40-60 μm) using eluent systems as described for each experiment.

All NMR spectra were recorded on Varian VNMRS 500 MHz, Jeol 400 MHz, or Bruker AV 400 MHz spectrometers. NMR data were processed using MNova 12.0.4 software. Proton and carbon-13 NMR spectra are reported as chemical shifts (δ) in parts per million (ppm) relative to residual undeuterated solvent peak or TMS. Coupling constants (*J*) are reported in units of hertz (Hz) and are rounded to the nearest 0.5 Hz for ^{1}H NMR and the nearest 1 Hz for ^{13}C NMR. The following abbreviations are used to describe multiplets: s (singlet), d (doublet), ad (apparent doublet), q (quartet), p (pentet), m (multiplet), br (broad). Structural assignments were made with additional information from gCOSY, gHSQC, and gHMBC experiments. HRMS measurements were carried out on Agilent 6530 Accurate-Mass Q-TOF LC/MS and Jeol Accutof GCX EI-TOF instruments. IR spectra were measured on a Perkin Elmer Spectrum One FT-IR with ATR attachment. Known compounds have been checked against literature references and only relevant analytical data are given.

Determination of Deuteration Incorporation. Deuterium incorporation was quantified by comparing the ^{1}H NMR integral intensity at the deuterated position with the starting material (see Fig. S1). ^{1}H NMR experiments were run with T1 relaxation times of 1 second and integral intensities were calibrated against hydrogen signals that did not undergo H/D-exchange.

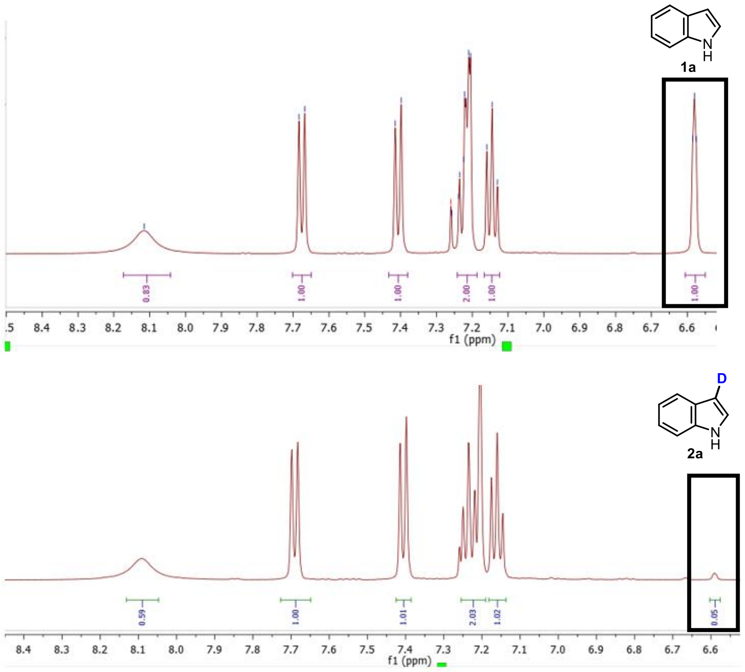

Figure S1: ^{1}H NMR spectra of unlabelled (top) and labelled/deuterated (bottom) indole in $CDCl_3$. Integration of the signal at 6.47 ppm decreases from 100% (**1a**) to 5% intensity (**2a**), indicating 95% deuterium incorporation.

REACTION INFORMATION

Reaction Optimisation

Table S1. Deuterium source & additive screen

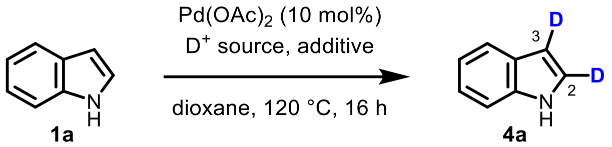

Entry	D^+ Source	Additive	C2 deuteration [%]	C3 deuteration [%]
**1**	CD_3CO_2D (1.14 mL)	-	40	52
**2**	CD_3CO_2D (1.14 mL)	NaOAc (1.5 equiv)	81	72
**3**[a]	CD_3CO_2D (0.6 mL)	NaOAc (1.5 equiv)	80	40
**4**[b]	**CD_3CO_2D (0.6 mL)**	**NaOAc (1.5 equiv)**	**80**	**70**
**5**	$\underline{CH_3}CO_2D$ (0.6 mL)	NaOAc (1.5 equiv)	37	65
**6**	CD_3CO_2D (0.6 mL)	NaOAc (4 equiv)	64	69
**7**	CD_3CO_2D (0.3 mL)	NaOAc (1.5 equiv)	70	25
**8**[c]	CD_3CO_2D (0.2 mL)	NaOAc (1.5 equiv)	51	14
**9**	D_2O (1 mL)	NaOAc (1.5 equiv)	42	87
**10**	D_2O (1 mL)	NaOAc (1.5 equiv), CH_3CO_2H (0.5 mL)	41	72
**11**	D_2O (1 mL)	NaOAc (1.5 equiv), pivalic anhydride (1.5 equiv)	44	43

Reaction conditions: **1a** (0.2 mmol), $Pd(OAc)_2$ (10 mol%), D^+ source, additive, 1,4-dioxane (1.5 mL, 0.13M), 120 °C, 16 h. Deuterium incorporation determined by ^1H NMR. [a] 1,4-Dioxane used without additional drying. [b] 1,4-Dioxane dried over molecular sieves (for the importance of anhydrous solvents in this reaction, see Table S5 below). [c] 0.8 mL dioxane (0.25M); 47% deuterium incorporation at C7.

The use of CH_3CO_2D significantly reduced deuteration at C2 compared with CD_3CO_2D (entry 5 cf. entry 4) – presumably due to proton exchange between the methyl and hydroxyl positions in acetic acid. Low C2-deuteration was also observed with D_2O (entry 9), and efforts to generate a viable deuterium source *in situ* from cheaper D_2O and CH_3CO_2H (entry 10) or pivalic anhydride (entry 11) were unsuccessful.

Table S2. Solvent, temperature & time screen

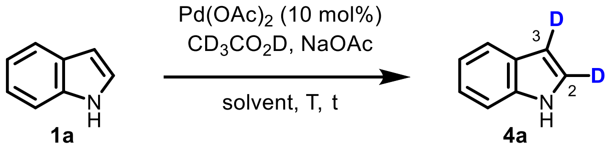

Entry	Solvent	Temperature [°C], time	C2 deuteration [%]	C3 deuteration [%]
**1**	dioxane	80 °C, 16 h	58	13
**2**	dioxane	120 °C, 16 h	81	72
**3**	dioxane	120 °C, 4 h	60	20
**4**	no solvent	120 °C, 16 h	60	63
**5**	MeCN	80 °C, 16 h	27	43

Reaction conditions: **1a** (0.2 mmol), $Pd(OAc)_2$ (10 mol%), CD_3CO_2D (0.6 mL), NaOAc (1.5 equiv.), solvent (1.5 mL, 0.13M), temperature, time. Deuterium incorporation determined by ^1H NMR.

Table S3. Catalyst screen

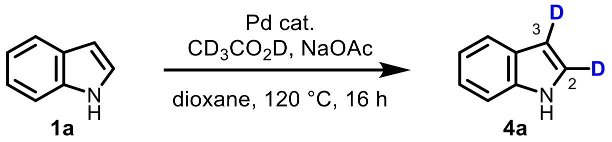

Entry	Pd cat.	C2 deuteration [%]	C3 deuteration [%]
**1**	$Pd(OAc)_2$ (10 mol%)	81	72
**2**	$Pd(OAc)_2$ (5 mol%)	15	47
**3**	none	0	50
**4**	$Pd(PPh_3)_4$ (10 mol%)	0	45

Reaction conditions: **1a** (0.2 mmol), Pd cat., CD_3CO_2D (0.6 mL), NaOAc (1.5 equiv.), 1,4-dioxane (1.5 mL, 0.13M), 120 °C, 16 h. Deuterium incorporation determined by ^1H NMR.

As the palladium loading is decreased, the uncatalyzed acid-base background reaction becomes dominant.

Table S4. Screening of reverse deuterium exchange conditions

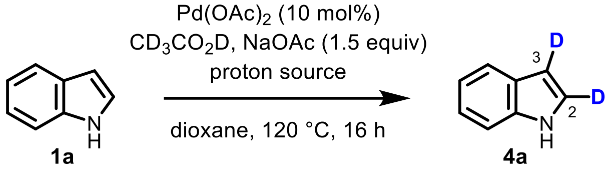

Entry	Proton source	C2 deuteration [%]	C3 deuteration [%]
**1**	-	80	70
**2**	NaOAc (15 equiv)	80	0
**3**[a]	CH_3CO_2H (10 equiv)	80	46
**4**[a]	silica gel (200 mg)	80	17
**5**	NaOH (50 equiv)	80	15
**6**	K_2CO_3 (1 equiv), $MeOH/H_2O$ (one pot)	24	25
**7**[a]	K_2CO_3 (1 equiv), $MeOH/H_2O$ (two steps)	81	0

Reaction conditions: **1a** (0.2 mmol), $Pd(OAc)_2$ (10 mol%), CD_3CO_2D (0.6 mL), NaOAc (1.5 equiv.), 1,4-dioxane (1.5 mL, 0.13M), 120 °C, 16 h. Deuterium incorporation determined by ^1H NMR. [a] Proton source added in second step, second step run at 80 °C, 16 h.

The Importance of Dry Solvents

Table S5. Effect of anhydrous solvent

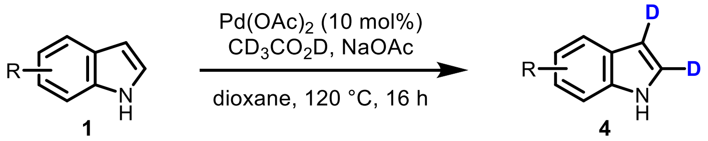

anhydrous dioxane	old (wet) dioxane	old (wet) dioxane + 4Å molecular sieves

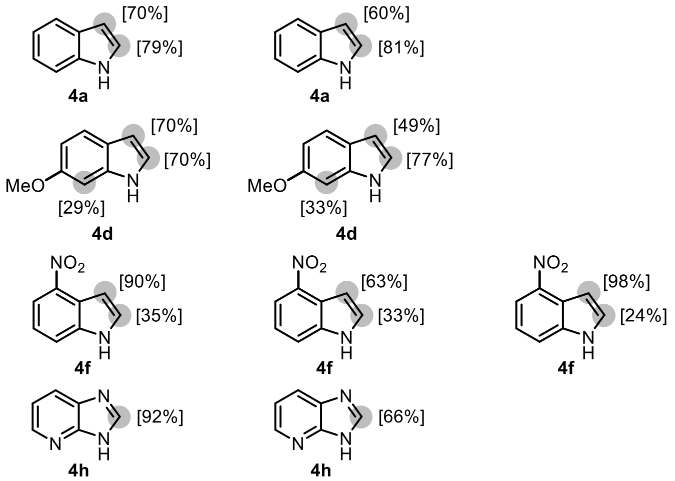

Conditions: **1** (0.4 mmol), $Pd(OAc)_2$ (10 mol%), NaOAc (1.5 equiv.), CD_3CO_2D/dioxane (1.2 mL/3 mL), 120 °C, 16 h. Grey circles show the labelling positions, with values in brackets denoting isotope incorporation, as determined by ^1H NMR, before purification on silica.

The use of anhydrous 1,4-dioxane and rigorous drying of NaOAc proved instrumental to avoid acid/base background reactions causing isotopic dilution at C3 (Table S5). When an older bottle of dioxane was used, deuterium incorporation at C3 was markedly lower than with a new bottle of anhydrous 1,4-dioxane (stored over molecular sieves). Pleasingly, the addition of 4Å molecular sieves to a reaction with the older dioxane largely succeeded in suppressing isotopic dilution (**4f** in Table S5).

Optimized Reaction Conditions

Method A: C2-Deuteration.

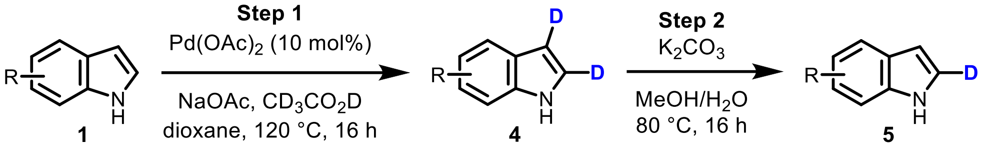

Step 1: Pd-catalysed C2- and C3-deuteration. To a mixture of indole **1** (0.4 mmol), $Pd(OAc)_2$ (9 mg, 10 mol%) and NaOAc (50 mg, 0.6 mmol) in anhydrous 1,4-dioxane (3 mL, 0.13 M) was added deuterated acetic acid (CD_3CO_2D) (1.2 mL, 10 mmol). The reaction was placed in an oil bath pre-heated to 120 °C and stirred for 16 h, after which it was allowed to cool to rt. The crude mixture was filtered over celite, and solvent was removed under vacuum. The compounds were purified via silica flash column chromatography.

Step 2: Treatment with base. d_2-Deuterated indole **4** (0.2 mmol) was dissolved in a solution of MeOH (1.2 ml) and H_2O (0.4 ml). K_2CO_3 (28 mg, 0.2 mmol) was added. The reaction was placed in an oil bath pre-heated to 80 °C and stirred for 16 h, after which it was allowed to cool to rt. The reaction was diluted with H_2O and extracted 3 times with DCM. The organic layer was dried using $MgSO_4$ and solvent was removed under reduced pressure to provide the pure product. In some cases, further purification by silica flash column chromatography was required.

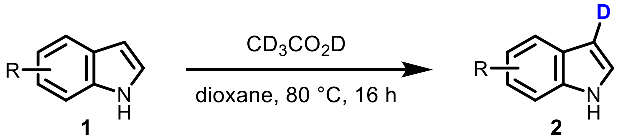

Method B: C3-Deuteration. To a mixture of indole **1** (0.2 mmol) in anhydrous 1,4-dioxane (0.6 mL, 0.3M) was added deuterated acetic acid (CD_3CO_2D) (1.2 mL, 10 mmol). The reaction was placed in an oil bath pre-heated to 80 °C and stirred for 16 h, after which it was cooled to room temperature and solvents were removed under vacuum. No further purification was required, unless stated otherwise.

CHARACTERIZATION OF COMPOUNDS

C2-Deuterated Indoles

2-Deuterio-1*H*-indole (5a)

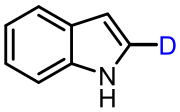

5a was synthesized from **4a** (24 mg, 0.2 mmol) according to method A, step 2 to give 19 mg (80% yield, 67% deuterium incorporation) of the title compound as an orange solid. No purification was required.

When the synthesis of **5a** was scaled up to 119 mg (1.0 mmol) following method A, step 2, 84 mg (71% yield, 60% deuterium incorporation) of the title compound were isolated.

Analytical data is provided for the 0.2 mmol scale reaction:

^{1}H NMR (400 MHz, $CDCl_3$) δ 8.17 (brs, 1H, N*H*), 7.65 (dd, *J* = 7.0, 1.0 Hz, 1H), 7.41 (dq, *J* = 8.0, 1.0 Hz, 1H), 7.22–7.17 (m, **1.33H**), 7.12 (ddd, *J* = 8.0, 7.0, 1.0 Hz, 1H), 6.56 (d, *J* = 2.0 Hz, 1H). Analytical data matches literature values.[1]

3-Methyl-1*H*-indole-2-*d* (5b)

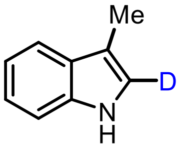

5b was synthesized from **4b** (26 mg, 0.2 mmol) according to method A, step 2. It was purified by silica flash column chromatography (gradient: cyclohexane to 7:3 cyclohexane:ethyl acetate) to afford 44 mg (83% yield, 80% deuterium incorporation) of the title compound as an off-white solid.

^{1}H NMR (500 MHz, $CDCl_3$) δ 7.87 (brs, 1H, N*H*), 7.59 (d, *J* = 8.0 Hz, 1H), 7.35 (d, *J* = 8.0 Hz, 1H), 7.22–7.18 (m, 1H), 7.15–7.11 (m, 1H), 6.98 (s, **0.2H**), 2.35 (s, 3H). Analytical data matches literature values.[2]

7-Methyl-1*H*-indole-2-*d* (5c)

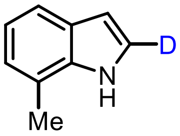

5c was synthesized from **4c** (26 mg, 0.2 mmol) according to method A, step 2 to yield 18 mg (68% yield, 55% deuterium incorporation) of the title compound as a brown solid. No purification was required.

^{1}H NMR (400 MHz, $CDCl_3$) δ 8.09 (brs, 1H, N*H*), 7.52 (d, *J* = 8.0 Hz, 1H), 7.22 (dd, *J* = 3.0, 2.5 Hz, **0.45H**), 7.08–6.99 (m, 2H), 6.60–6.55 (m, 1H), 2.51 (s, 3H); **^{13}C{^{1}H} NMR** (101 MHz, $CDCl_3$) δ 135.5, 127.5, 123.9, 122.6, 120.3, 120.1, 118.6, 103.3, 16.8; **FT-IR (neat)** ν 3412, 2900, 2852, 1750, 1595, 1452, 1377, 1340, 1245, 1072, 803, 783, 745, 698, 666 cm^{-1}; **HRMS** not detected by ESI (positive / negative), APCI or EI.

6-Methoxy-1*H*-indole-2,7-d_2 (5d)

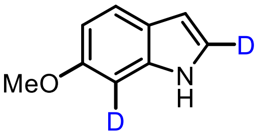

5d was synthesized from **4d** (29 mg, 0.2 mmol) according to method A, step 2 to afford 21 mg (71% yield, 65% deuterium incorporation at C2, 25% deuterium incorporation at C7) of the title compound as a pink solid. No purification was required.

^{1}H NMR (400 MHz, $CDCl_3$) δ 8.04 (s, 1H), 7.52 (d, *J* = 8.5 Hz, 1H), 7.10 (dd, *J* = 3.0, 2.5 Hz, **0.35H**), 6.88 (d, *J* = 2.0 Hz**, 0.75H**), 6.83–6.80 (m, 1H), 6.49 (d, *J* = 2.0 Hz, 1H), 3.85 (s, 3H); **^{13}C{^{1}H} NMR** (101 MHz, $CDCl_3$) δ 156.5, 136.6, 123.1, 122.2, 121.4, 110.0, 102.4, 94.6, 55.8; **FT-IR (neat)** ν 3389, 3009, 2961, 2835, 2528, 1620, 1579, 1501, 1455, 1463, 1290, 1161,

1111, 1058, 1026, 954, 810, 422, 661 cm^{-1}; **HRMS** not detected by ESI (positive / negative), APCI or EI.

4-Nitro-1*H*-indole-2-*d* (5f)

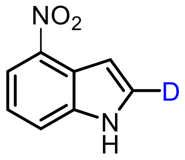

5f was synthesized from **4f** (32 mg, 0.2 mmol) according to method A, step 2 to yield 22 mg (67% yield, 65% deuterium incorporation) of the title compound as a yellow solid. No purification was required.

^{1}H NMR (600 MHz, CD_3OD) δ 8.08 (dd, *J* = 8.0, 1.0 Hz, 1H), 7.80 (dt, *J* = 8.0, 1.0 Hz, 1H), 7.57 (d, *J* = 3.0 Hz, **0.35H**), 7.26 (t, *J* = 8.0 Hz, 1H), 7.19–7.07 (m, 1H); **^{13}C{^{1}H} NMR** (151 MHz, CD_3OD) δ 139.9, 138.6, 129.2, 121.7, 119.7, 118.1, 116.5, 101.4; **FT-IR (neat)** ν 3310, 2918, 1560, 1530, 1450, 1306, 1220, 770, 650 cm^{-1}; **HRMS** not detected by ESI (positive / negative), APCI or EI.

2-(1*H*-Indol-3-yl-2-*d*)ethan-1-ol / tryptophol-2-*d* (5g)

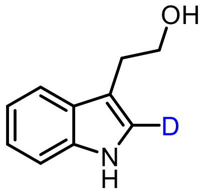

5g was synthesized from tryptophol (64 mg, 0.4 mmol) according to method A, step 1. It was purified by silica flash column chromatography (gradient: 100% cyclohexane to 100% ethyl acetate) to afford 25 mg (39% yield, 60% deuterium incorporation) of the title compound as a beige solid.

^{1}H NMR (500 MHz, $CDCl_3$) δ 8.02 (brs, 1H, N*H*), 7.64 (d, *J* = 8.0 Hz, 1H), 7.37 (d, *J* = 8.0 Hz, 1H), 7.23–7.18 (m, 1H), 7.16–7.11 (m, 1H), 7.05 (s, **0.4H**), 4.36 (t, *J* = 6.5 Hz, 2H), 3.10 (t, *J* = 7.0 Hz, 2H); **^{13}C{^{1}H} NMR** (126 MHz, $CDCl_3$) δ 136.3, 127.6, 122.3, 122.1, 119.6, 118.9, 112.3, 111.3, 64.7, 24.9; **FT-IR (neat)** ν 3404, 3056, 2955, 2922, 2855, 1724, 1448, 1257, 1076, 984, 745 cm^{-1}; **HRMS** not detected by ESI (positive / negative), APCI or EI.

1*H*-Benzoimidazole-2-*d* (5h)

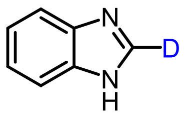

Method A, step 1 (Pd catalysis): **5h** was synthesized from benzimidazole (47 mg, 0.4 mmol). The crude product was purified by silica flash column chromatography (gradient: cyclohexane to 1:1 cyclohexane:ethyl acetate) to afford 45 mg (94% yield, 92% deuterium incorporation) of the title compound as a white solid.
Method B (Pd-free): **5h** was synthesized from benzimidazole (24 mg, 0.2 mmol) to afford 23 mg (97% yield, 96% deuterium incorporation).

Method A: **^{1}H NMR** (500 MHz, CD_3OD) δ 8.15 (s, **0.08H**), 7.64–7.57 (m, 2H), 7.28–7.23 (m, 2H). Method B: **^{1}H NMR** (500 MHz, CD_3OD) δ 8.18 (s, **0.04H**), 7.62–7.59 (m, 2H), 7.26–7.24 (m, 2H). Analytical data matches literature values.[3]

_N_-Fmoc-2-deuterotryptophan / (((9_H_-fluoren-9-yl)methoxy)carbonyl)tryptophan-2-_d_ (5k)

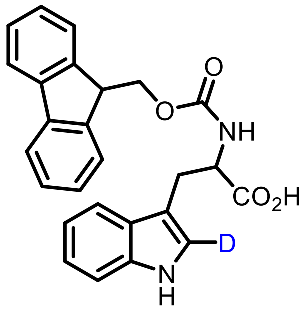

5k was synthesized from Fmoc-Trp(Boc)-OH (171 mg, 0.4 mmol) according to method A, step 1. It was purified by silica flash column chromatography (gradient: ethyl acetate to 9:1 ethyl acetate:methanol) to afford 141 mg (83% yield, 56% deuterium incorporation) of the title compound as a beige solid.

^{1}H NMR (500 MHz, $CDCl_3$) δ 8.05 (s, 1H, N*H*), 7.76 (d, *J* = 7.5 Hz, 2H), 7.61 (d, *J* = 8.0 Hz, 1H), 7.53 (t, *J* = 9.0 Hz, 2H), 7.44 – 7.33 (m, 3H), 7.31–7.27 (m, 2H), 7.21 (t, *J* = 7.5 Hz, 1H), 7.13 (t, *J* = 7.5 Hz, 1H), 6.96 (s, **0.44H**), 5.32 (d, *J* = 8.0 Hz, 1H), 4.77 (d, *J* = 8.0 Hz, 1H), 4.46–4.34 (m, 2H), 4.19 (t, *J* = 7.0 Hz, 1H), 3.36 (s, 1H); **^{13}C{^{1}H} NMR** (126 MHz, $CDCl_3$) δ 175.5, 156.0, 143.7, 141.3 (2C), 136.1, 127.7, 127.1, 125.1, 122.3, 119.98, 119.96, 119.90, 118.6, 111.3 (2C), 67.1, 54.5, 47.1, 27.6; **FT-IR (neat)** ν 3361, 3060, 1717, 1664, 1512, 1450, 1412, 1335, 1224, 1139, 1083, 1048, 905, 758 cm^{-1}; **HRMS** (ESI+) *m/z* calc. for $C_{26}H_{21}DN_2O_4$ $[M+H]^+$ 428.1715, found 428.1726.

1_H_-Idol-2,3,5,6,7-d_5-4-ol (5m)

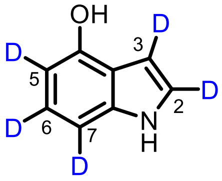

5m was synthesized from 4-hydroxyindole (53 mg, 0.4 mmol) according to method A, step 1. Deuterium incorporation was determined by crude ^{1}H NMR using trimethoxybenzene (22 mg, 0.13 mmol, 0.33 equiv.) as internal standard: 89% at deuterium incorporation at C2, ca. 84% at C3*, 85% at C5, 75% at C6, ca. 84% at C7* (*signals for H_3 and H7 overlap, and an average value is given). Peak assignment was carried out by comparison with ^{1}H NMR and COSY spectra of un-deuterated 4-hydroxyindole.

^{1}H NMR (500 MHz, CD_3OD) δ 7.08 (s, **0.25H**, H_6), 6.90 (s, **0.32H**, H_3+H_7), 6.51 (d, *J* = 3.0 Hz, **0.15H,** H_5), 6.37–6.39 (m, **0.11H,** H_2).

C2-and-C3-Deuterated Indoles

Note: While every effort was made to *keep the compounds on silica for the minimum amount of time possible* to avoid protonation (isotopic dilution) at C3, all compounds in this section showed a reduction in deuteration during column chromatography (compared to the crude product). *E.g.* for compound **4a**, deuterium incorporation was 70% at C3 prior to purification, but dropped to 45% after silica flash column chromatography (see Table S5 above for more C3 deuteration values before silica flash column chromatography).

1*H*-Indole-2,3-d_2 (4a)

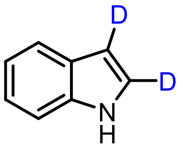

4a was synthesized from indole (47 mg, 0.4 mmol) according to method A, step 1. It was purified by silica flash column chromatography (gradient: cyclohexane to 7:3 cyclohexane:ethyl acetate) to afford 40 mg (83% yield, 81% deuterium incorporation at C2, 45% deuterium incorporation at C3) of the title compound as an orange solid.

When the synthesis of **4a** was scaled up to 234 mg (2.0 mmol) following a modified method A, step 1 (reaction time = 32 h), 145 mg (61% yield, 65% deuterium incorporation at C2, 76% deuterium incorporation at C3) of the title compound was isolated after purification by column chromatography (gradient: cyclohexane to 7:3 cyclohexane:ethyl acetate).

Analytical data is provided for the 0.4 mmol reaction:

^{1}H NMR (500 MHz, $CDCl_3$) δ 8.13 (brs, 1H, N*H*), 7.67 (d, *J* = 8.0 Hz, 1H), 7.41 (d, *J* = 8.0 Hz, 1H), 7.23–7.19 (m, **1.19H**), 7.14 (at, *J* = 7.5 Hz, 1H), 6.57 (s, **0.55H**); **^{13}C{^{1}H} NMR** (126 MHz, $CDCl_3$) δ 135.9, 128.0, 124.2, 122.1, 120.9, 119.9, 111.1, 102.8; **FT-IR (neat)** ν 3400, 3053, 2917, 2849, 1698, 1615, 1454, 1335, 1243, 1092, 1009, 931, 710, 669 cm^{-1}; **HRMS** not detected by ESI (positive / negative), APCI or EI.

7-Methyl-1*H*-indole-2,3-d_2 (4c)

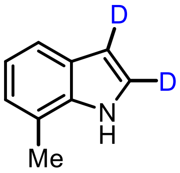

4c was synthesized from 7-methylindole (52 mg, 0.4 mmol) according to method A, step 1. It was purified by silica flash column chromatography (gradient: cyclohexane to 7:3 cyclohexane:ethyl acetate) to afford 48 mg (90% yield, 75% deuterium incorporation at C2, 45% deuterium incorporation at C3) of the title compound as an off-white solid.

^{1}H NMR (500 MHz, $CDCl_3$) δ 8.08 (brs, 1H, N*H*), 7.52 (d, *J* = 8.0 Hz, 1H), 7.23–7.21 (m, **0.25H**), 7.06 (t, *J* = 7.5 Hz, 1H), 7.01 (dd, *J* = 7.5, 1.0 Hz, 1H), 6.58–6.57 (m, **0.55H**), 2.52 (s, 3H). Analytical data matches literature values.[4]

6-Methoxy-1*H*-indole-2,3,7-d_3 (4d)

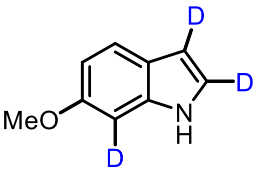

4d was synthesized from 6-methoxyindole (59 mg, 0.4 mmol) according to method A, step 1. It was purified by silica flash column chromatography (gradient: cyclohexane to 7:3 cyclohexane:ethyl acetate) to afford 45 mg (58% yield, 80% deuterium incorporation at C2, 45% deuterium incorporation at C3, 30% deuterium incorporation at C7) as an off-white solid.

^{1}H NMR (500 MHz, $CDCl_3$) δ 8.01 (brs, 1H, N*H*), 7.53 (d, *J* = 8.5 Hz, 1H), 7.09 (s, **0.2H**), 6.88 (s, **0.7H**), 6.82 (d, *J* = 8.5 Hz, 1H), 6.49 (s, **0.55H**), 3.86 (s, 3H); **^{13}C{^{1}H} NMR** (126 MHz, $CDCl_3$) δ 156.4, 136.5, 122.9, 122.1, 121.2, 109.9, 102.5, 94.5, 55.7; **FT-IR (neat)** ν 3391, 3042, 3007, 2959, 2921, 2835, 1617, 1499, 1451, 1441, 1427, 1388, 1346, 1289, 1160, 1026, 810, 663 cm^{-1}; **HRMS** not detected by ESI (positive / negative), APCI or EI.

4-Fluoro-1*H*-indole-2,3-*d*$_2$ (4e)

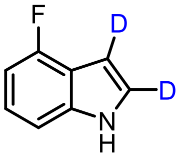

4e was synthesized from 4-fluoroindole (54 mg, 0.4 mmol) according to method A, step 1. It was purified by silica flash column chromatography (gradient: cyclohexane to 7:3 cyclohexane:ethyl acetate) to afford 41 mg (75% yield, 85% deuterium incorporation at C2, 75% deuterium incorporation at C3) of the title compound as a brown oil.

^{1}H NMR (500 MHz, $CDCl_3$) δ 8.24 (s, 1H), 7.19 (ad, *J* = 8.0 Hz, **1.15H**), 7.14 – 7.09 (m, 1H), 6.80 (dd, *J* = 10.0, 8.0 Hz, 1H), 6.65 (s, **0.25H**); **^{13}C{^{1}H} NMR** (101 MHz, $CDCl_3$) δ 156.6 (d, *J* = 247 Hz), 138.5 (d, *J* = 12 Hz), 124.1, 122.6 (d, *J* = 8.0 Hz), 117.2 (d, *J* = 23 Hz), 107.2, 104.6 (d, *J* = 19 Hz), 98.7; **^{19}F NMR** (470 MHz, $CDCl_3$) δ −122.06 – −122.20 (m); **FT-IR (neat)** ν 3396, 2922, 2852, 1723, 1629, 1578, 1504, 1466, 1438, 1402, 1349, 1226, 1034, 768 cm^{-1}; **HRMS** not detected by ESI (positive / negative), APCI or EI.

4-Nitro-1*H*-indole-2,3-*d*$_2$ (4f)

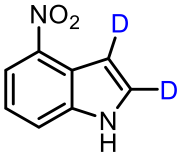

4f was synthesized from 4-nitroindole (65 mg, 0.4 mmol) according to a modified method A, step 1, where the reaction time was extended to 32 h. It was purified by silica flash column chromatography (gradient: cyclohexane to 7:3 cyclohexane:ethyl acetate) to afford 45 mg (69% yield, 65% deuterium incorporation at C2, 91% deuterium incorporation at C3) of the title compound as a yellow solid. (After 16 h, only 32% deuterium incorporation at C2 and 90% deuterium incorporation at C3 were observed.)

^{1}H NMR (400 MHz, CD_3OD) δ 8.08 (dd, *J* = 8.0, 1.0 Hz, 1H), 7.81 (dd, *J* = 8.0, 1.0 Hz, 1H), 7.58 (s, **0.35H**), 7.26 (t, *J* = 8.0 Hz, 1H), 7.15–7.13 (m, **0.09H**); **^{13}C{^{1}H} NMR** (126 MHz, CD_3OD) δ 141.3, 140.1, 130.6, 123.2, 121.2, 119.5, 117.9, 102.9; **FT-IR (neat)** ν 3313, 2956, 2917, 2849, 1560, 1503, 1413, 1359, 1303, 1252, 1099, 1059, 811, 748 cm^{-1}; **HRMS** not detected by ESI (positive / negative), APCI or EI.

7-Aza-1*H*-indole-2,3-*d*$_2$ (4i)

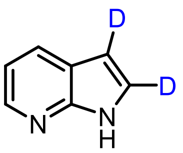

4i was synthesized from 7-azaindole.
Method A, step 1 (47 mg, 0.4 mmol) followed by purification on silica flash column chromatography (gradient: cyclohexane to 7:3 cyclohexane:ethyl acetate) afforded 27 mg (57% yield, 11% deuterium incorporation at C2, 90% deuterium incorporation at C3) of the title compound as an off-white solid.
A modified method A, step 2 (conditions applied twice) did not lead to complete protonation at C3, instead affording 20 mg (83% yield, 8% deuterium incorporation at C2, 6% deuterium incorporation at C3) of the title compound as an off-white solid. Analytical data is provided for the product from method A, step 1:

^{1}H NMR (500 MHz, $CDCl_3$) δ 11.44 (brs, 1H, N*H*), 8.36 (d, J = 4.5 Hz, 1H), 7.98 (d, J = 8.0 Hz, 1H), 7.40 (s, **0.89H**), 7.11 (dd, J = 8.0, 4.5 Hz, 1H), 6.52 (d, J = 3.5 Hz, **0.10H**); **^{13}C{^{1}H} NMR** (126 MHz, CDCl3) δ 148.9, 142.5, 129.2, 125.3, 120.6, 115.9, 100.8; **FT-IR (neat)** ν 3670, 2969, 2904, 1599, 1563, 1500, 1496, 1417 1329, 1278, 1111, 913, 885, 747 cm^{-1}; **HRMS** not detected by ESI (positive / negative), APCI or EI.

Pindolol-2,3-*d*$_2$ (4l)

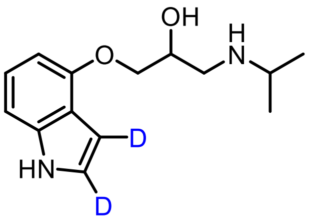

4l was synthesized from pindolol (99 mg, 0.4 mmol) according to method A, step 1. It was purified by silica flash column chromatography (gradient: 100% cyclohexane to 100% ethyl acetate) to afford 70 mg (70% yield, 57% deuterium incorporation at C2, 34% deuterium incorporation at C3) of the title compound as an off-white solid. Method A, step 2 did not lead to a reduction of deuterium incorporation at C3.

^{1}H NMR (400 MHz, CD_3OD) δ 7.14 (d, J = 3.0 Hz, **0.43H**), 7.08–6.97 (m, 2H), 6.57–6.54 (m, 1H), 6.52 (d, J = 7.0 Hz, **0.66H**), 4.36–4.28 (m, 1H), 4.20 (dd, J = 10.0, 5.0 Hz, 1H), 4.11 (dd, J = 10.0, 6.0 Hz, 1H), 3.41 (p, J = 6.5 Hz, 1H), 3.16 (dd, J = 12.5, 9.5 Hz, 1H), 1.35 (dd, J = 6.5, 4.0 Hz, 6H), (one of the proton peaks is obscured by the CD_3COD solvent peak); **^{13}C{^{1}H} NMR** (101 MHz, CD_3OD) δ 153.1, 139.2, 124.2, 122.9, 120.0, 106.4, 101.2, 99.2, 70.9, 67.2, 51.8, 48.9, 19.5, 19.1; **FT-IR (neat)** ν 3210, 3116, 2973, 2472, 2330, 1637, 1550, 1504, 1471, 1400, 1351, 1269, 1284, 1248, 1228, 1092, 740 cm^{-1}; **HRMS** (ESI+) *m/z* calc. for $C_{14}H_{19}D_2N_2O_2$ $[M+H]^+$ 251.1723, found 251.1718.

C3-Deuterated Indoles

1*H*-Indole-3-*d* (2a)

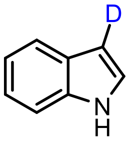

2a was synthesized from indole (23 mg, 0.2 mmol) according to method B to afford 24 mg (quantitative yield, 95% deuterium incorporation) of the title compound as an orange solid.

^{1}H NMR (500 MHz, $CDCl_3$) δ 7.97 (brs, 1H, N*H*), 7.57 (d, J = 8.0 Hz, 1H), 7.29 (d, J = 8.0 Hz, 1H), 7.14–7.08 (m, 2H), 7.04 (t, J = 7.5 Hz, 1H), 6.47 (d, J = 3.0 Hz, **0.05H**). Analytical data matches literature values.[5]

7-Methyl-1*H*-indole-3-*d* (2c)

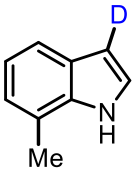

2c was synthesized from 7-methylindole (26 mg, 0.2 mmol) according to method B to afford 26 mg (quantitative yield, 94% deuterium incorporation) of the title compound as an off-white solid.

^{1}H NMR (500 MHz, $CDCl_3$) δ 8.04 (brs, 1H, N*H*), 7.56 (d, J = 8.0 Hz, 1H), 7.21 (s, 1H), 7.10 (t, J = 7.5 Hz, 1H), 7.05 (d, J = 7.0 Hz, 1H), 6.61 (d, J = 3.0 Hz, **0.06H**), 2.53 (s, 3H); **^{13}C{^{1}H} NMR** (126 MHz, $CDCl_3$) δ 135.3, 127.3, 123.6, 122.5, 120.2, 120.0, 118.5, 103.0, 16.7; **FT-IR (neat)** ν 3388, 3063, 2948, 2904, 2845, 1676, 1615, 1478, 1458, 1425, 1339, 1107, 782 cm^{-1}; **HRMS** not detected by ESI (positive / negative), APCI or EI.

6-Methoxy-1*H*-indole-3,7-*d*$_2$ (2d)

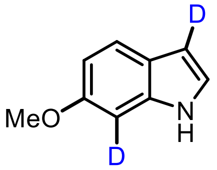

2d was synthesized from 7-methylindole (29 mg, 0.2 mmol) according to method B to afford 29 mg (97% yield, 95% deuterium incorporation at C3, 27% deuterium incorporation at C7) of the title compound an off-white solid.

^{1}H NMR (500 MHz, $CDCl_3$) δ 8.02 (brs, 1H, N*H*), 7.51 (d, *J* = 8.5 Hz, 1H), 7.10 (s, **0.73H**), 6.89 (d, *J* = 2.5 Hz, 1H), 6.81 (dd, *J* = 8.5, 2.5 Hz, 1H), 6.48 (d, *J* = 3.0 Hz, **0.05H**), 3.85 (s, 3H); **^{13}C{^{1}H} NMR** (126 MHz, $CDCl_3$) δ 156.6, 136.7, 123.0, 122.8, 122.2, 121.4, 110.0, 94.6, 55.8. Analytical data matches literature values.[5]

4-Fluoro-1*H*-indole-3-*d* (2e)

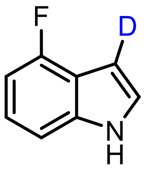

2e was synthesized from 4-fluoroindole (27 mg, 0.2 mmol) according to method B to afford 27 mg (quantitative yield, 93% deuterium incorporation) of the title compound as a brown oil.

^{1}H NMR (500 MHz, $CDCl_3$) δ 8.25 (brs, 1H, N*H*), 7.20–7.16 (m, 2H), 7.11 (td, *J* = 8.0, 5.0 Hz, 1H), 6.80 (dd, *J* = 10.5, 8.0 Hz, 1H), 6.65 (t, *J* = 2.5 Hz, **0.07H**); **^{13}C{^{1}H} NMR** (126 MHz, $CDCl_3$) δ 156.6 (d, *J* = 247 Hz), 138.6 (d, *J* = 11 Hz), 124.1, 122.6 (d, *J* = 8 Hz), 117.2 (d, *J* = 23 Hz), 107.2, 104.7 (d, *J* = 19 Hz), 98.9; **^{19}F NMR** (470 MHz, $CDCl_3$) δ −122.07 – −122.13 (m); **FT-IR (neat)** ν 3363, 2918, 2850, 1560, 1419, 1226, 1030, 873, 710, 662 cm^{-1}; **HRMS** not detected by ESI (positive / negative), APCI or EI.

4-Nitro-1*H*-indole-3-*d* (2f)

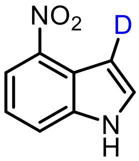

2f was synthesized from 4-nitroindole (32 mg, 0.2 mmol) according to method B to afford 32 mg (97% yield, 74% deuterium incorporation) of the title compound as a yellow solid.

^{1}H NMR (500 MHz, CD_3OD) δ 8.07 (d, *J* = 8.0 Hz, 1H), 7.81 (d, *J* = 8.0 Hz, 1H), 7.57 (s, 1H), 7.25 (t, *J* = 8.0 Hz, 1H), 7.13 (d, *J* = 3.0 Hz, **0.26H**); **^{13}C{^{1}H} NMR** (126 MHz, CD_3OD) δ 139.8, 138.6, 129.2, 121.8, 119.8, 118.2, 116.5, 101.4; **FT-IR (neat)** ν 3297, 2912, 1708, 1496, 1466, 1351, 1314, 1296, 1238, 1105, 1066, 986, 820, 715 cm^{-1}; **HRMS** not detected by ESI (positive / negative), APCI or EI.

7-Aza-1*H*-indole-3-*d* (2i)

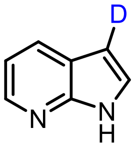

2i was synthesized from 7-azaindole (24 mg, 0.2 mmol) according to method B to afford 24 mg (quantitative yield, 98% deuterium incorporation) of the title compound as a brown solid.

^{1}H NMR (500 MHz, $CDCl_3$) δ 11.37 (brs, 1H, N*H*), 8.34 (dd, *J* = 5.0, 1.5 Hz, 1H), 7.98 (dd, *J* = 8.0, 1.5 Hz, 1H), 7.39 (s, 1H), 7.10 (dd, *J* = 8.0, 5.0 Hz, 1H), 6.52 (d, *J* = 3.5 Hz, **0.02H**). Analytical data matches literature values.[4]

Pindolol-3-*d* (2l)

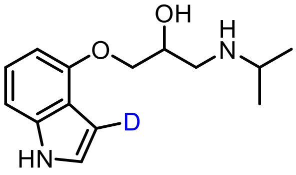

2l was synthesized from pindolol (50 mg, 0.2 mmol) according to method B to afford 49 mg (98% yield, 86% deuterium incorporation) of the title compound as a white solid.

^{1}H NMR (400 MHz, CD_3OD) δ 7.13 (d, *J* = 3.0 Hz, 1H), 7.07–6.96 (m, 2H), 6.55 (d, *J* = 3.0 Hz, 1H), 6.50 (d, *J* = 2.9 Hz, **0.14H**), 4.36–4.29 (m, 1H), 4.20 (dd, *J* = 10.0, 5.0 Hz, 1H), 4.10 (dd, *J* = 10.0, 6.0 Hz, 1H), 3.41 (p, *J* = 6.5 Hz, 1H), 3.33 (ad, *J* = 3.0 Hz, 1H), 3.17 (dd, *J* = 12.5, 9.5 Hz, 1H), 1.35 (dd, *J* = 6.5, 5.0 Hz, 6H); **^{13}C{^{1}H} NMR** (101 MHz, CD_3OD**)** δ 153.1, 139.3, 124.2, 122.8, 120.1, 106.4, 101.2, 99.5, 70.9, 67.0, 51.9, 48.7, 19.4, 18.8; **FT-IR (neat)** ν 3310, 3115, 2973, 2924, 2689, 2470, 2330, 1550, 1471, 1400, 1321, 1229, 1092, 929, 739 cm^{-1}; **HRMS** (ESI+) *m/z* calc. for $C_{14}H_{20}DN_2O_2$ $[M+H]^+$ 250.1661, found 250.1660.

References

[1] Liu, X.; Huang, J.; Xu, H.; Zhang, D.; Sun, Q.; He, L. Copper-Catalyzed Synthesis of 2-Aminocarbazoles through Cascade C–C and C–N Bond Formation and Aromatization. *Eur. J. Org. Chem.* **2019**, *2019* (5), 900–906. DOI: 10.1002/ejoc.201801297

[2] Pieters, G.; Taglang, C.; Bonnefille, E.; Gutmann, T.; Puente, C.; Berthet, J. C.; Dugave, C.; Chaudret, B.; Rousseau, B. Regioselective and Stereospecific Deuteration of Bioactive Aza Compounds by the Use of Ruthenium Nanoparticles. *Angew. Chemie Int. Ed.* **2014**, *53* (1), 230–234. DOI: 10.1002/anie.201307930

[3] Thapa, P.; Hazoor, S.; Chouhan, B.; Vuong, T. T.; Foss Jr., F. W. Flavin Nitroalkane Oxidase Mimics Compatibility with NOx/TEMPO Catalysis: Aerobic Oxidization of Alcohols, Diols, and Ethers. *J. Org. Chem.* **2020**, *85* (14), 9096–9105. DOI: 10.1021/acs.joc.0c01013

[4] Dong, B.; Cong, X.; Hao, N. Silver-catalyzed regioselective deuteration of (hetero)arenes and α-deuteration of 2-alkyl azaarenes. *RSC Adv.* **2020**, *10* (43), 25475–25479. DOI: 10.1039/d0ra02358b

[5] Darshana, D.; Sureram, S.; Mahidol, C.; Ruchirawat, S.; Kittakoop, P. Spontaneous conversion of prenyl halides to acids: application in metal-free preparation of deuterated compounds under mild conditions. *Org. Biomol. Chem.* **2021**, *19* (34), 7390–7402. DOI: 10.1039/d1ob01275d

NMR Spectra

C2-Deuterated Indoles

^{1}H NMR spectrum of (5a) (0.2 mmol scale)

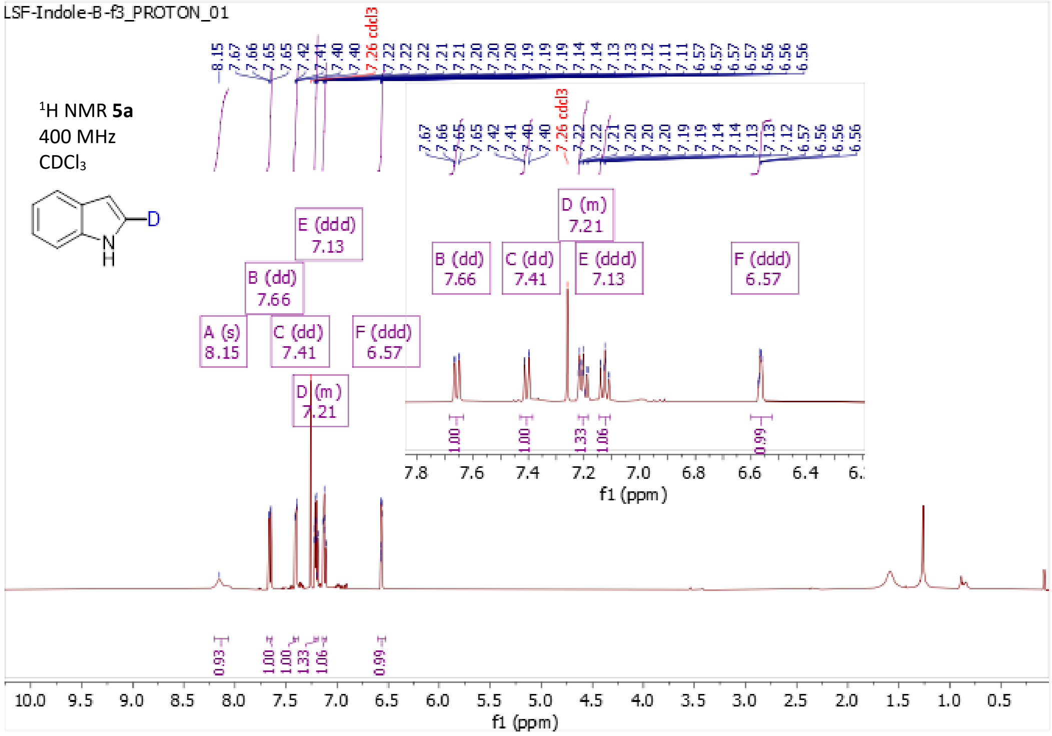

^{1}H NMR spectrum of (5a) (1.0 mmol scale)

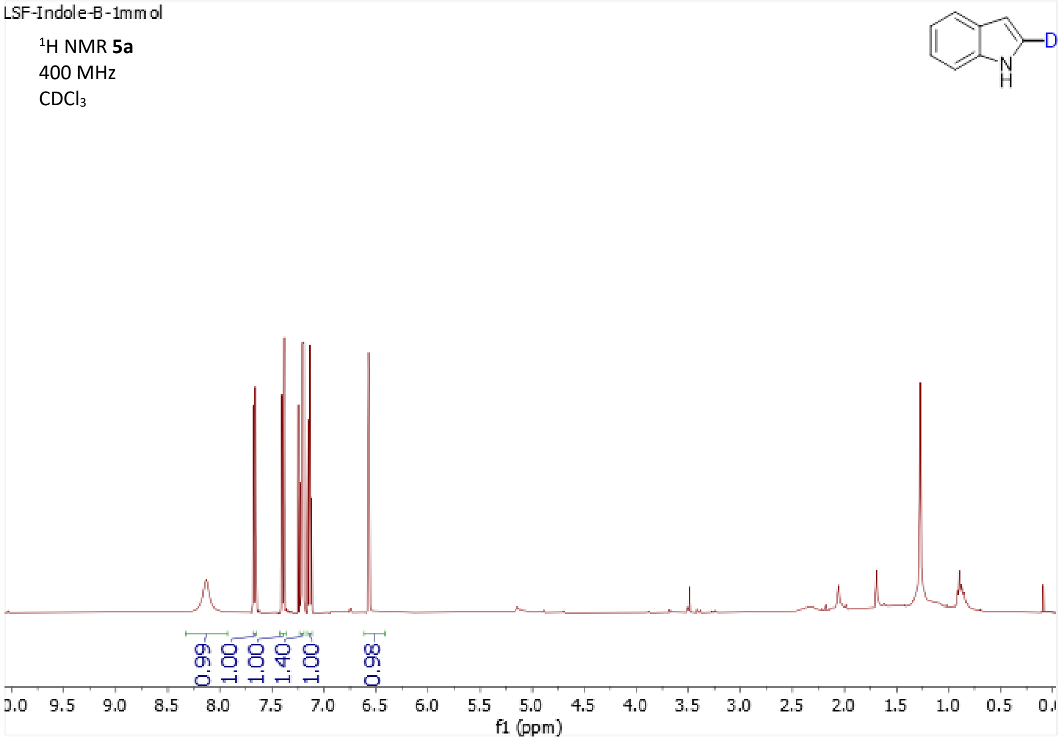

^{1}H NMR spectrum of (5b)

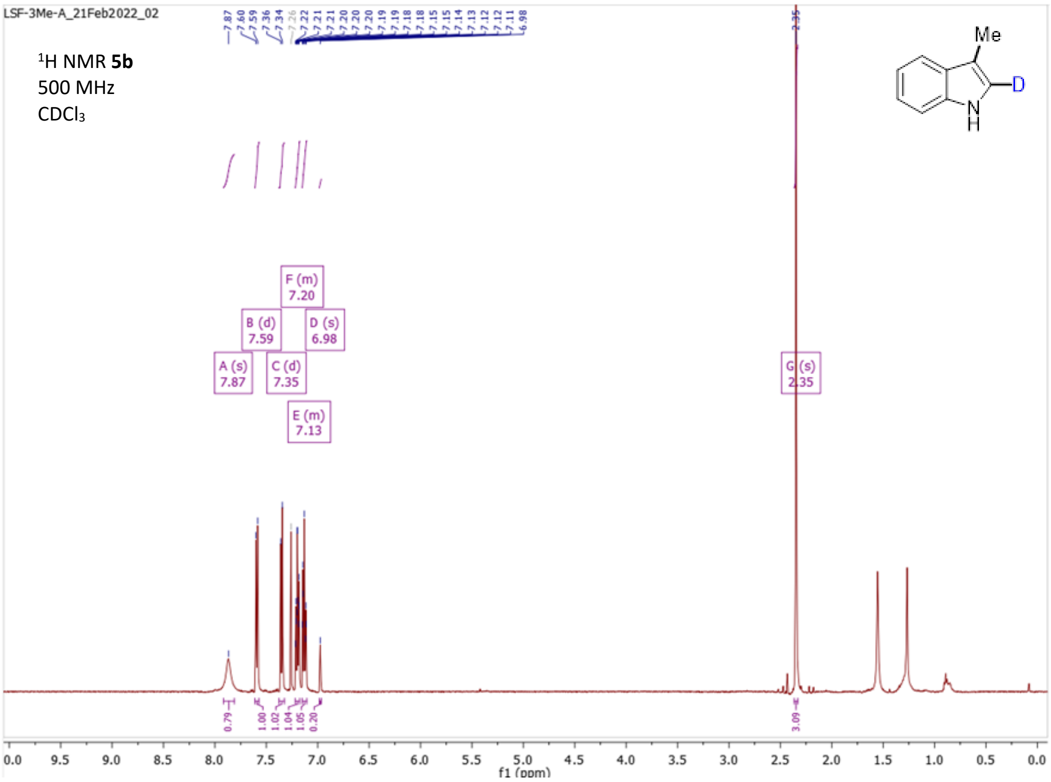

^{1}H NMR spectrum of (5c)

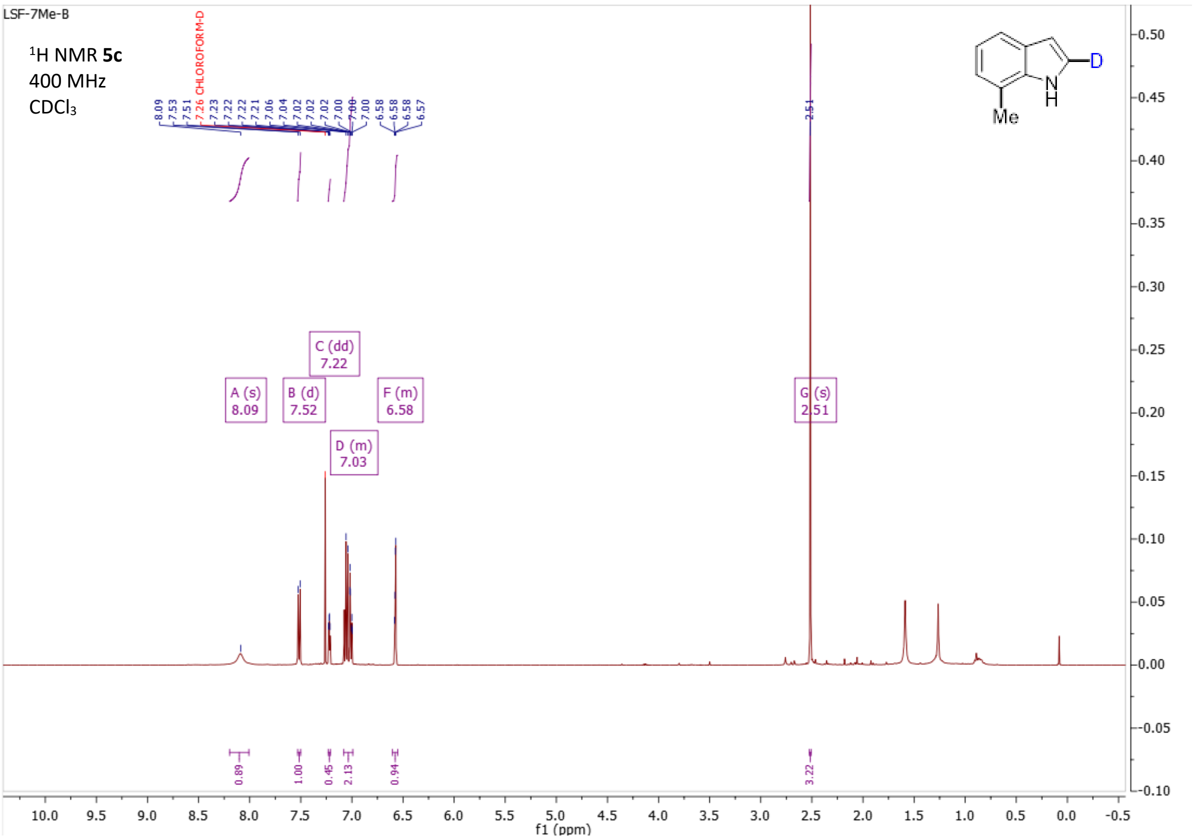

^{13}C NMR spectrum of (5c)

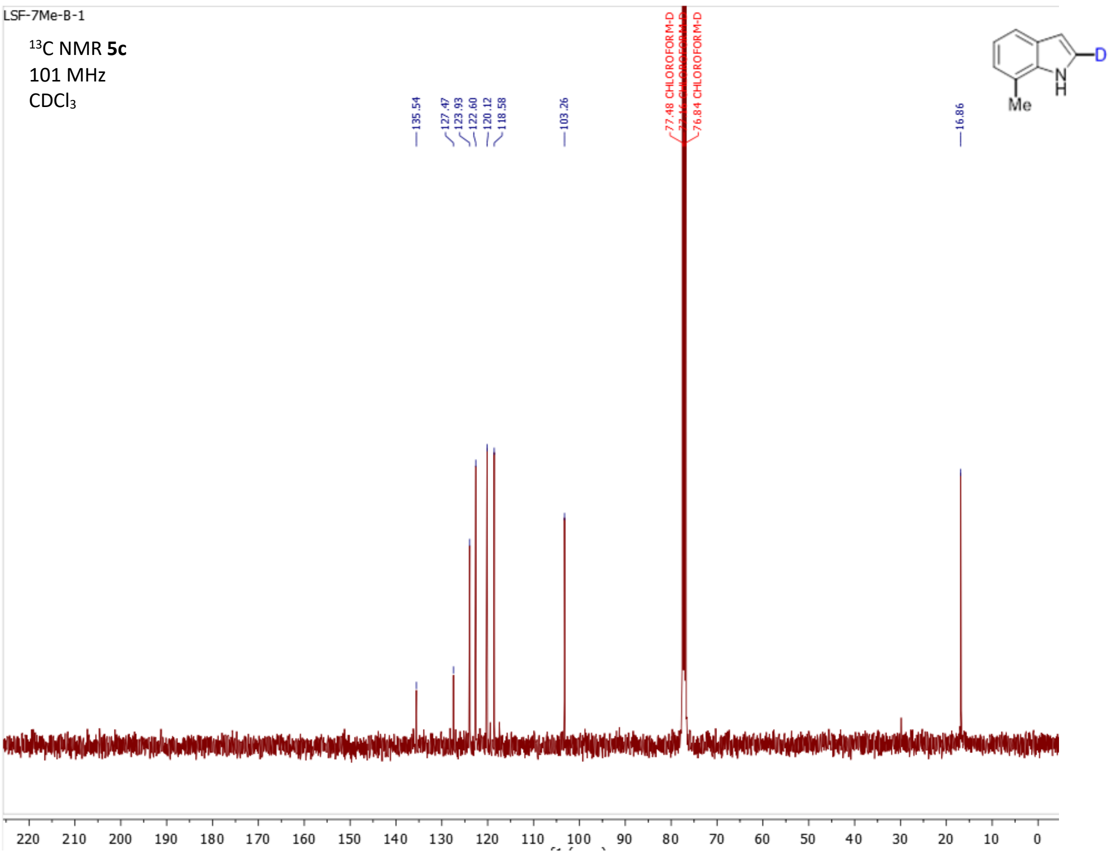

^{1}H NMR spectrum of (5d)

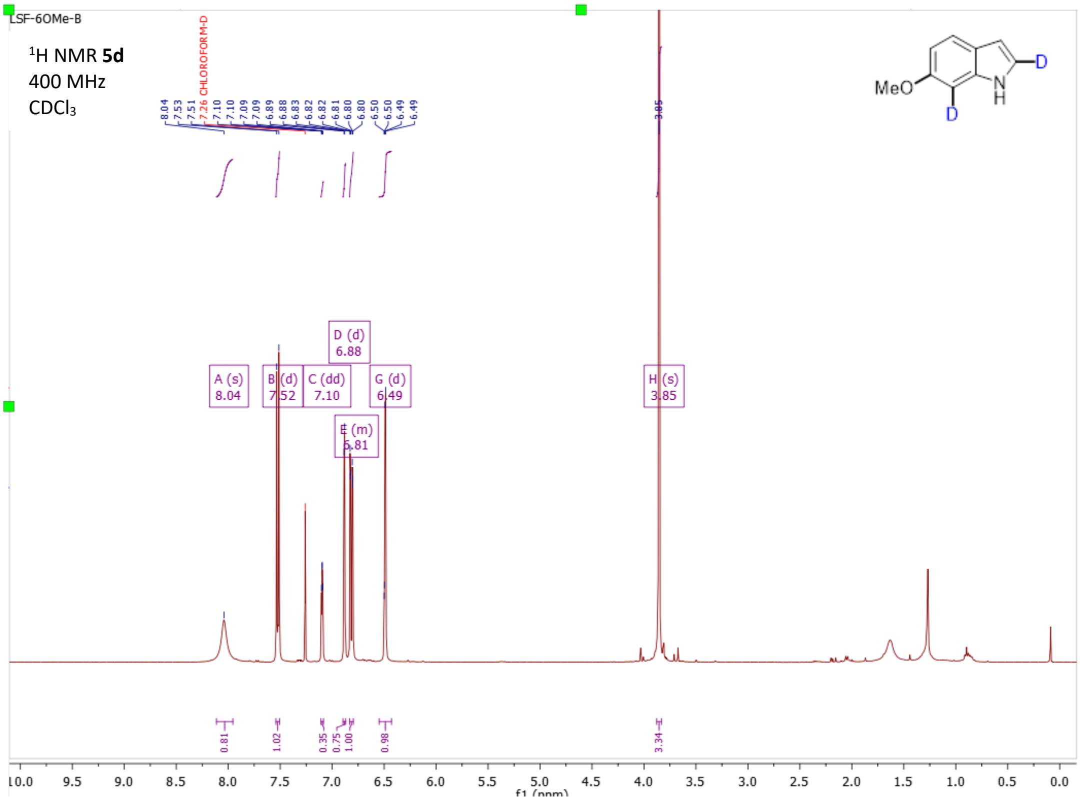

^{13}C NMR spectrum of (5d)

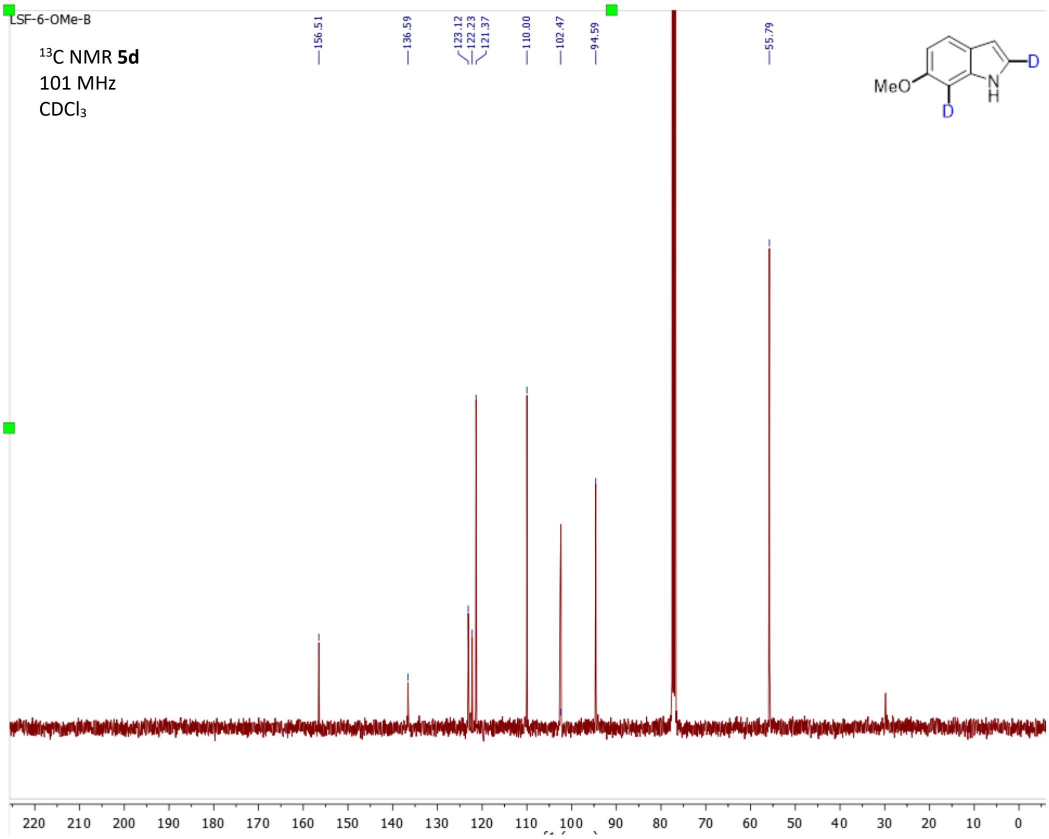

^{1}H NMR spectrum of (5f)

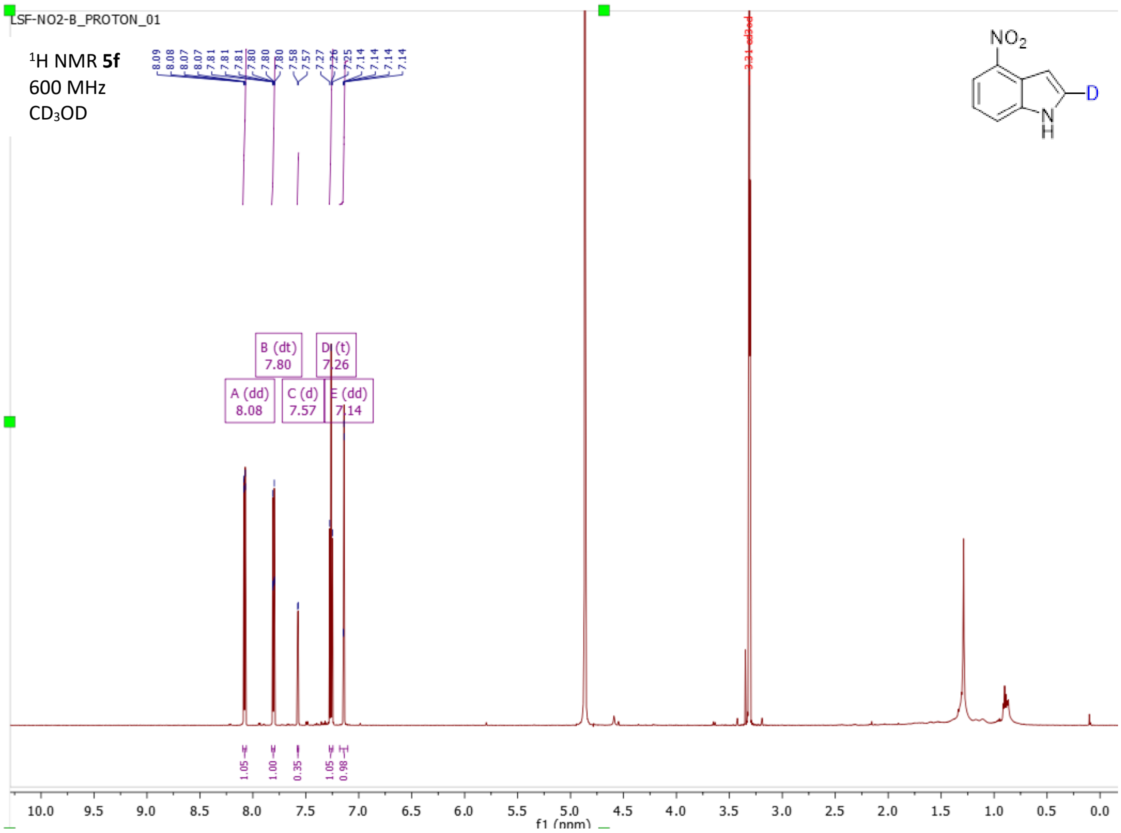

^{13}C NMR spectrum of (5f)

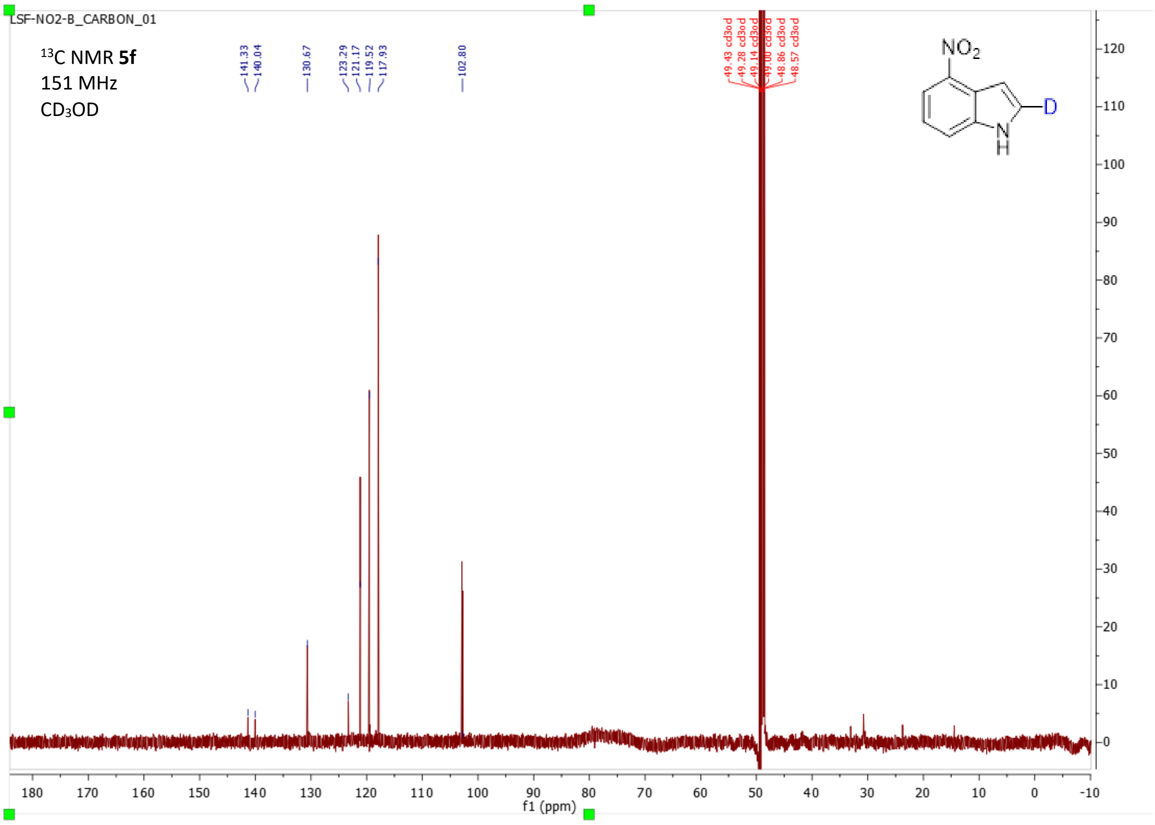

^{1}H NMR spectrum of (5g)

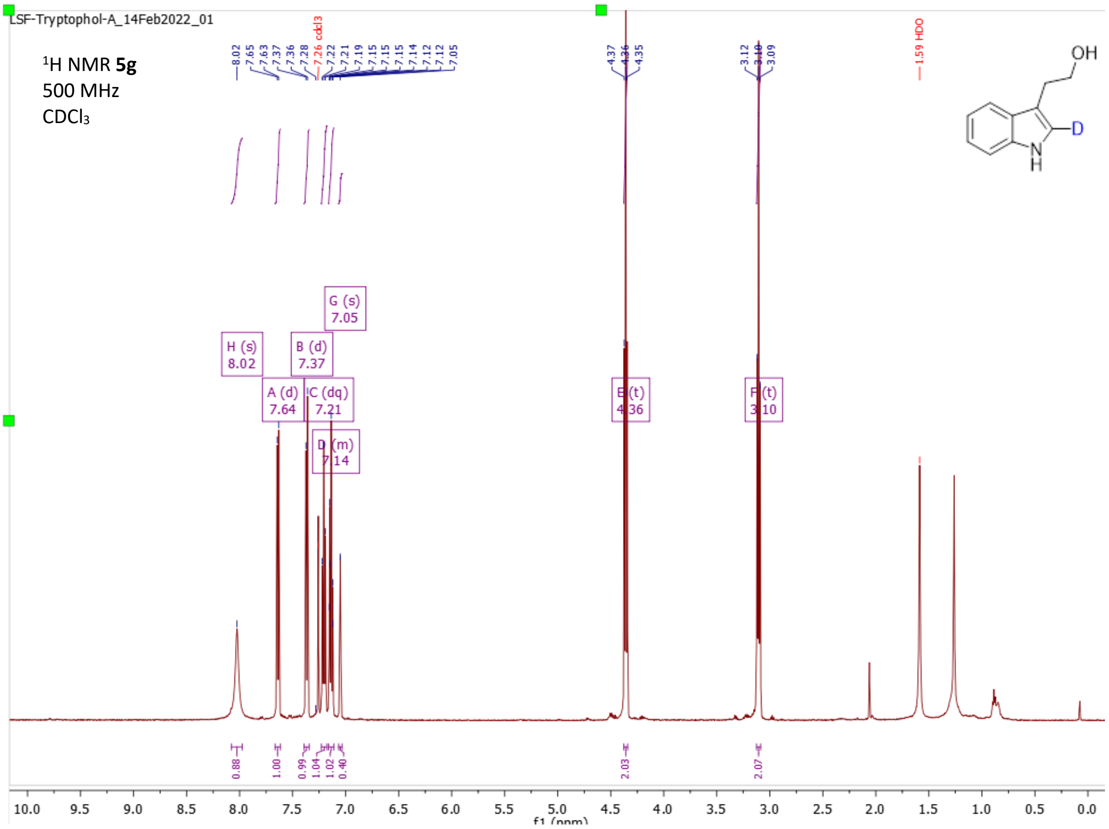

^{13}C NMR spectrum of (5g)

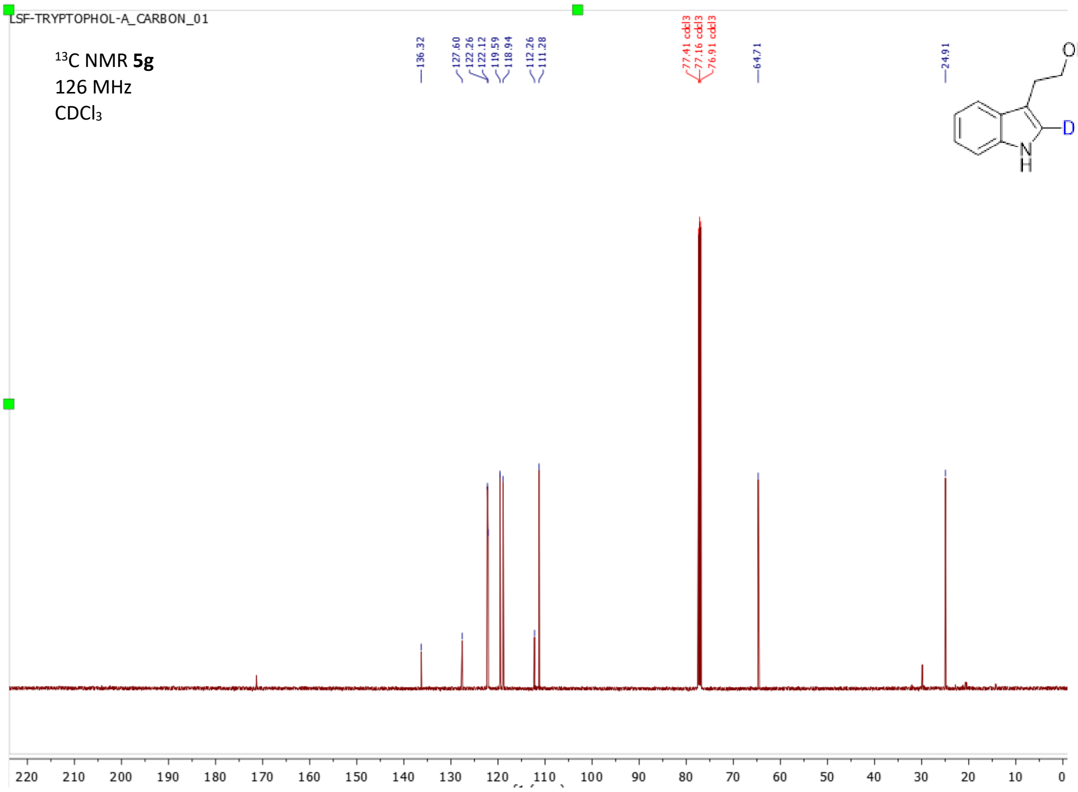

^{1}H NMR spectrum of (5h – method A)

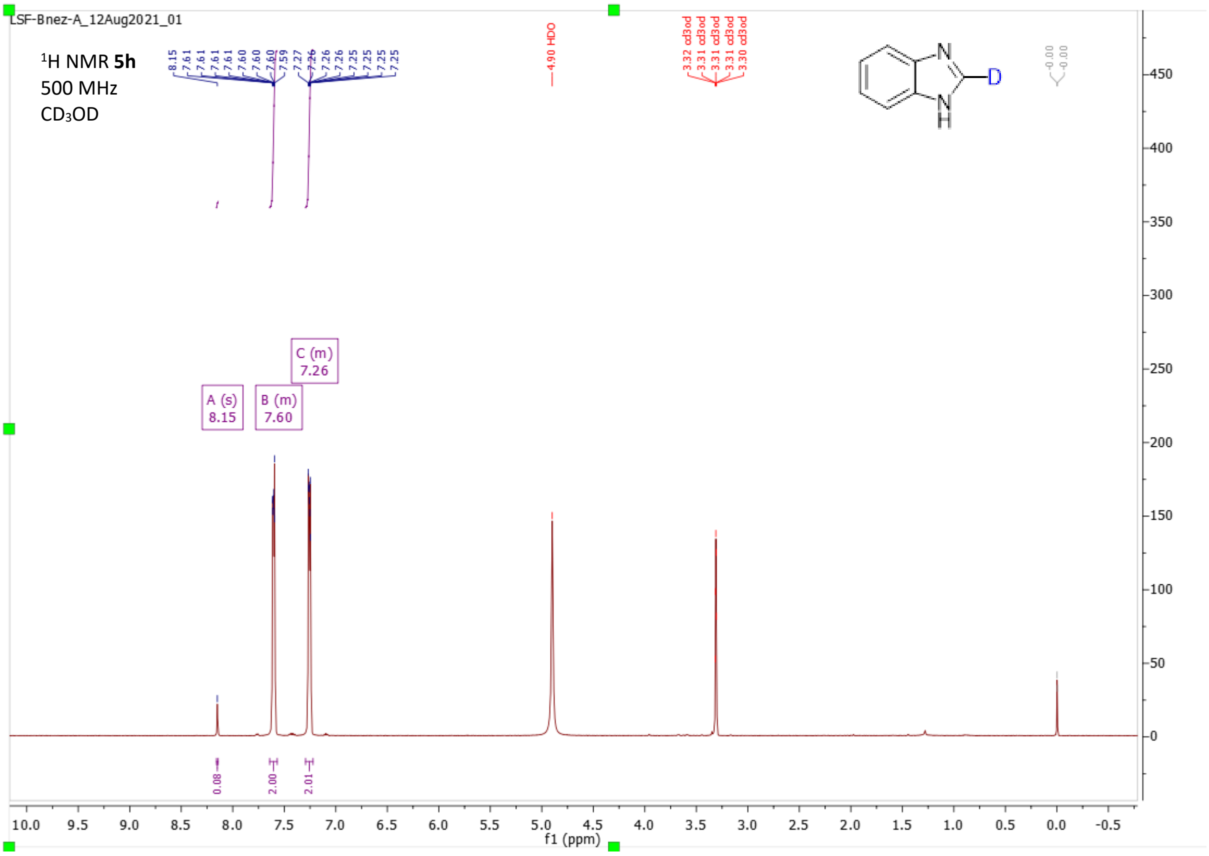

^{1}H NMR spectrum of (5h – method B)

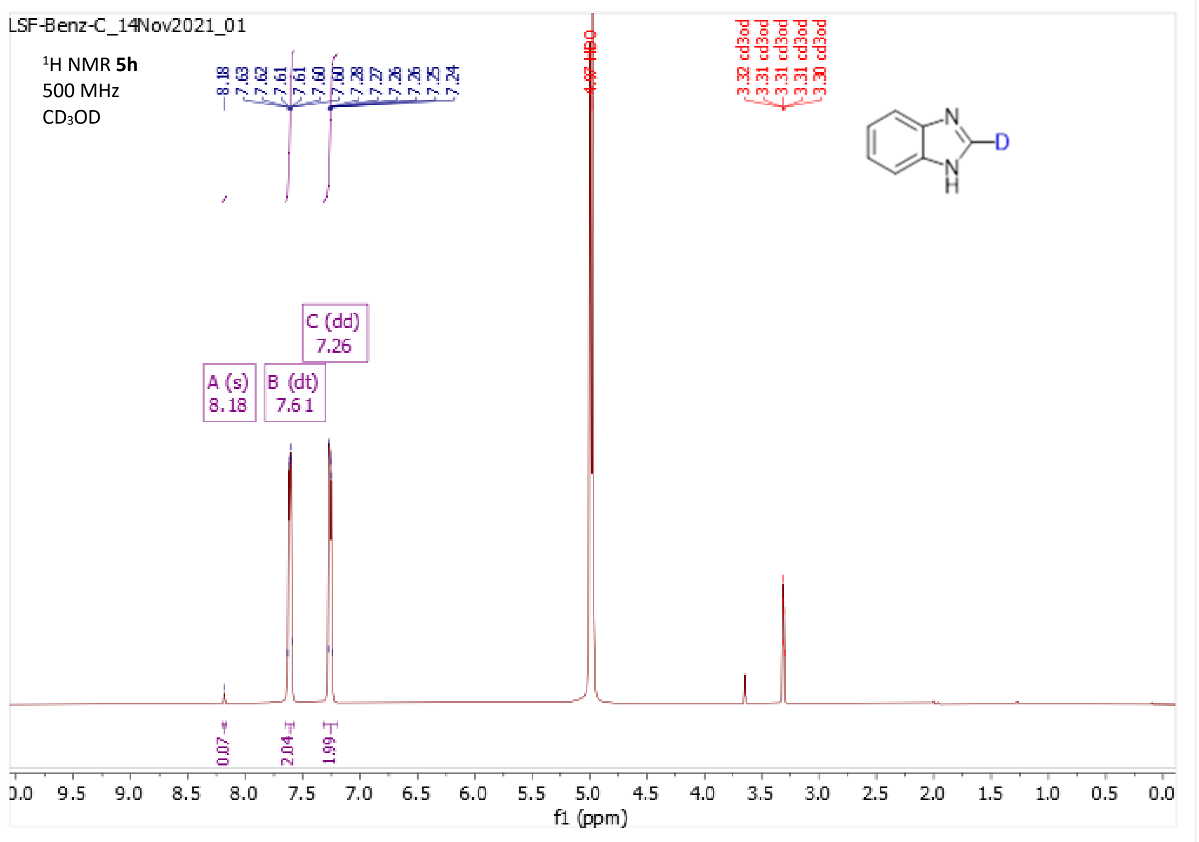

^{1}H NMR spectrum of (5k)

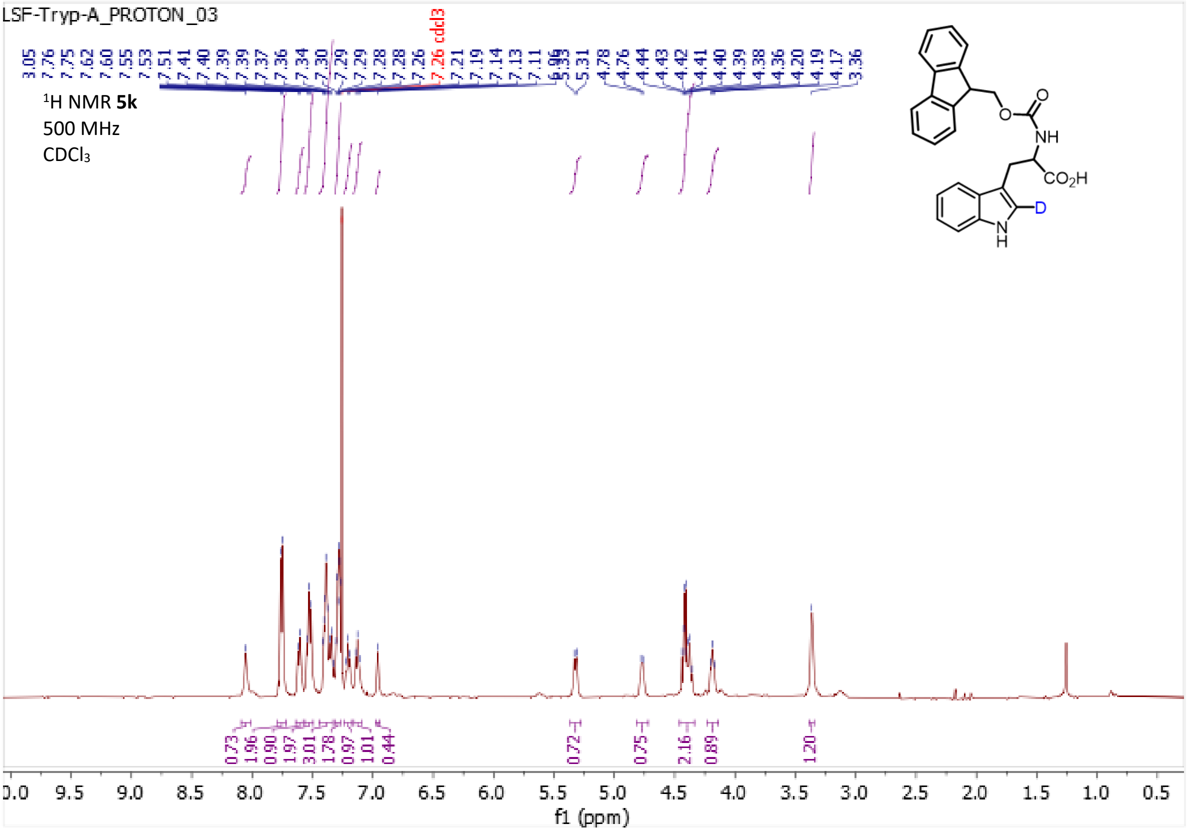

^{13}C NMR spectrum of (5k)

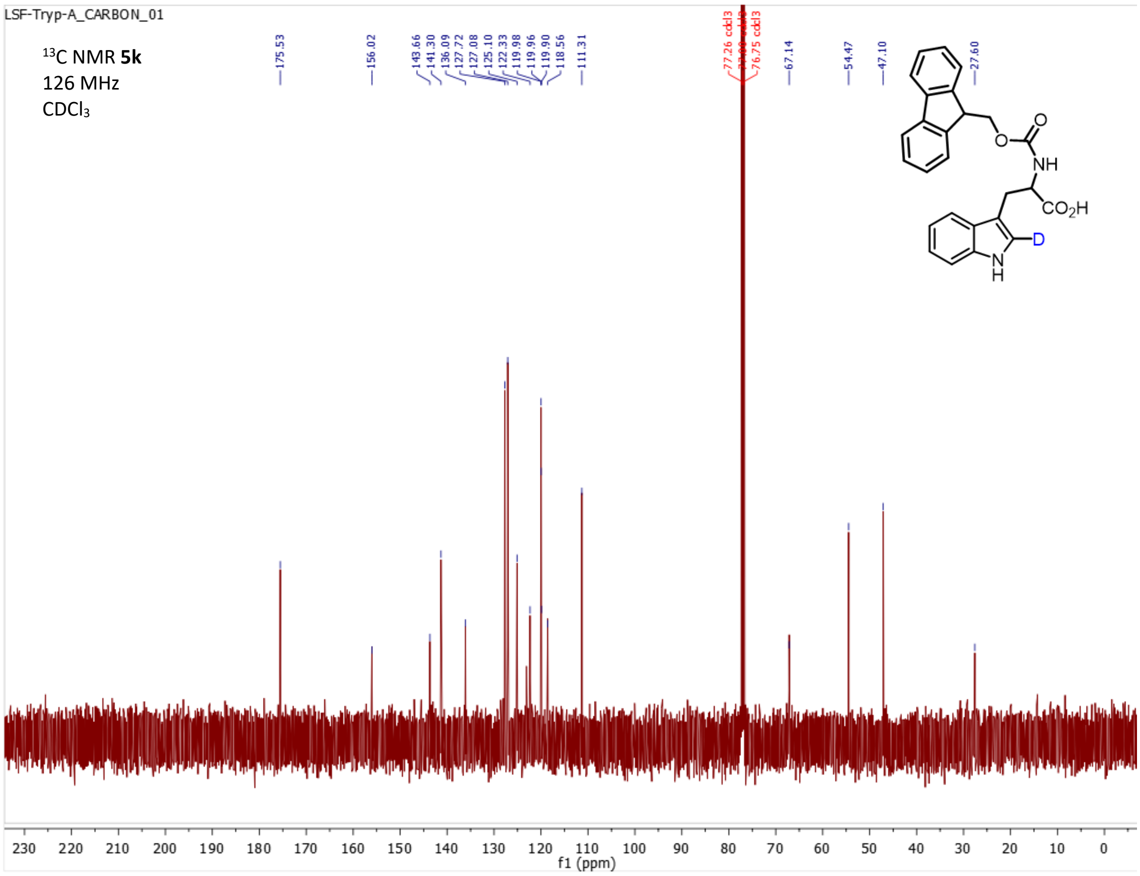

Crude ^{1}H NMR of (5m) + 0.3 equiv. trimethoxybenzene

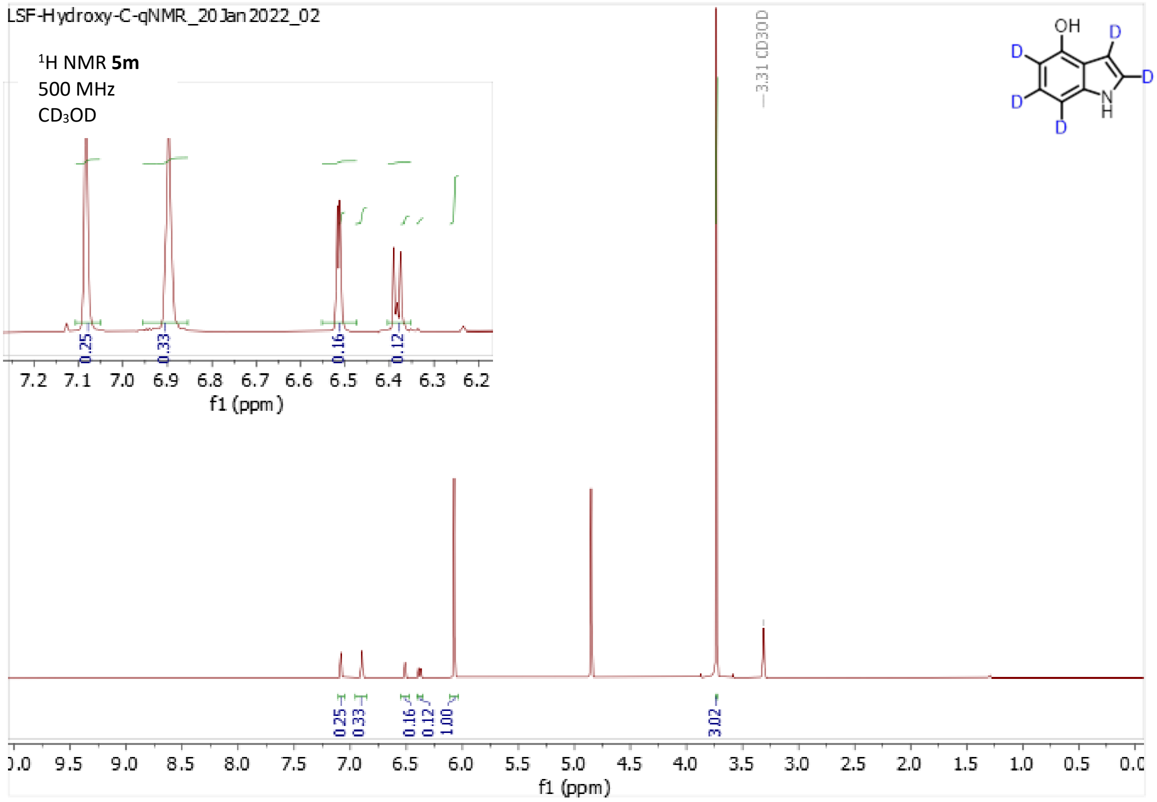

^{1}H NMR of un-deuterated 4-hydroxyindole (top) and deuterated (5m) (bottom)

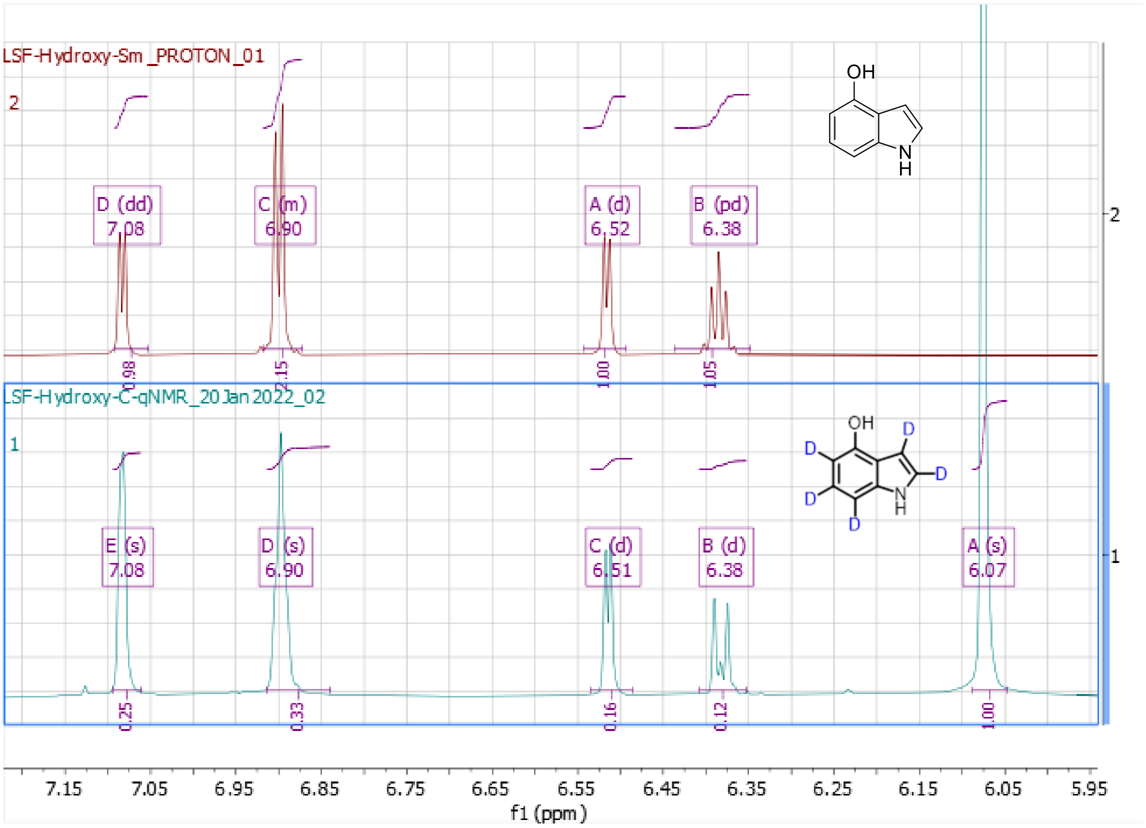

^{1}H NMR of un-deuterated 4-hydroxyindole

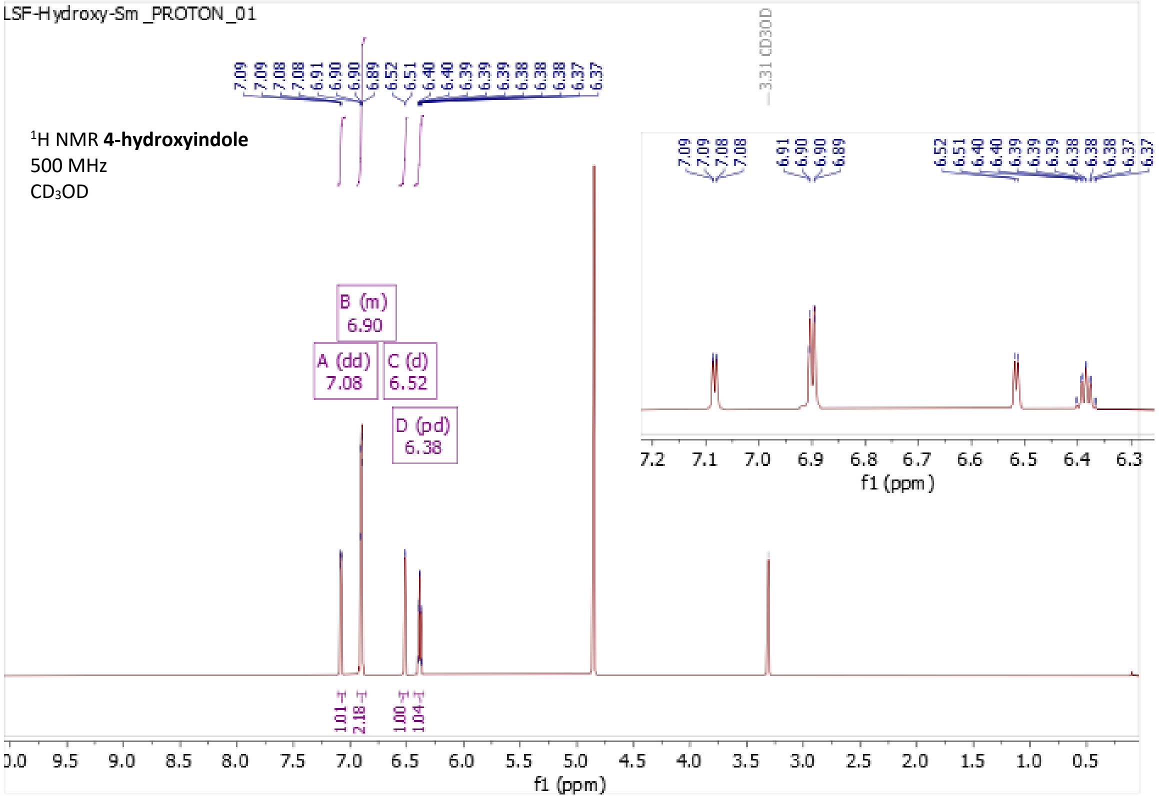

COSY of un-deuterated 4-hdyroxyindole

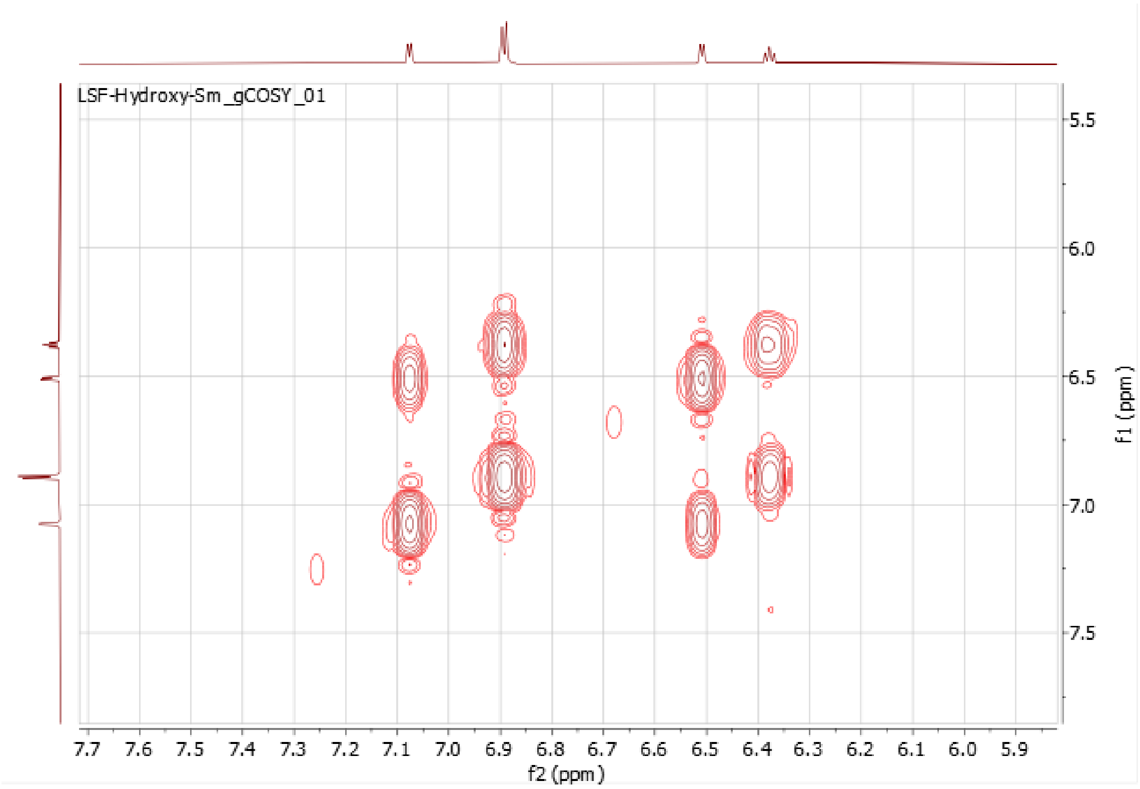

C2- and C3-Deuterated Indoles

^{1}H NMR spectrum of (4a) (0.4 mmol scale)

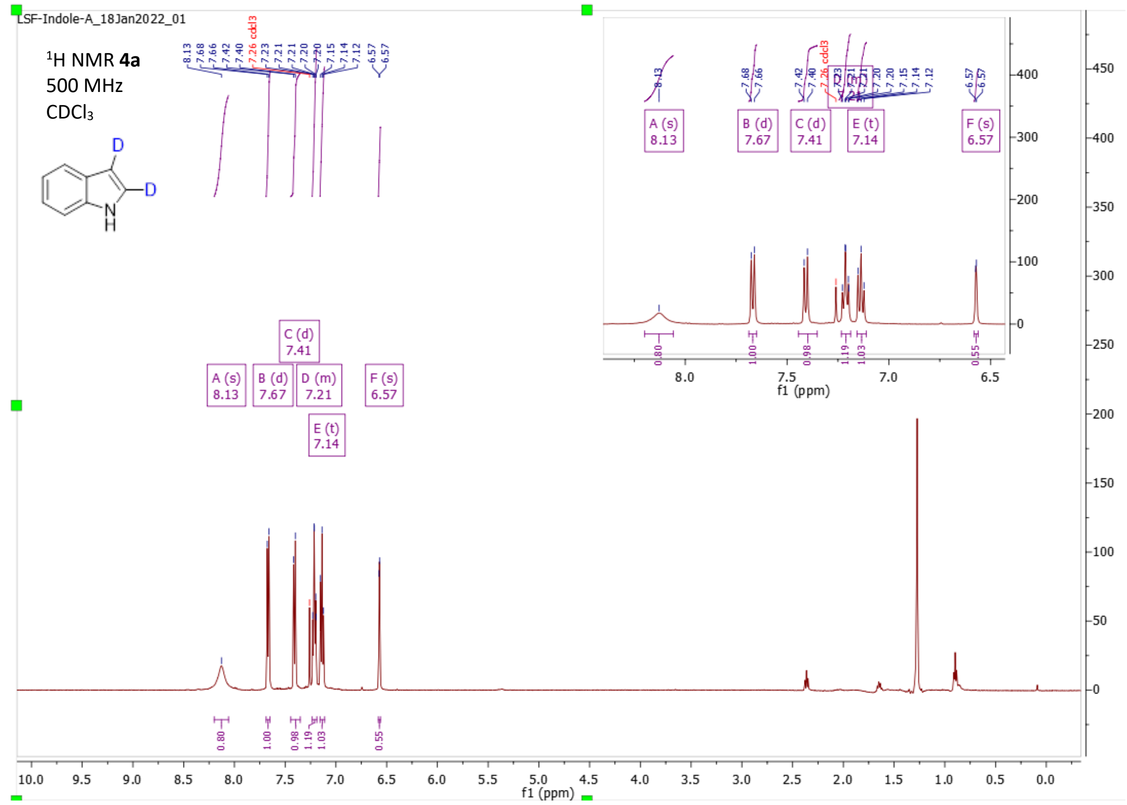

^{1}H NMR spectrum of (4a) (2.0 mmol scale)

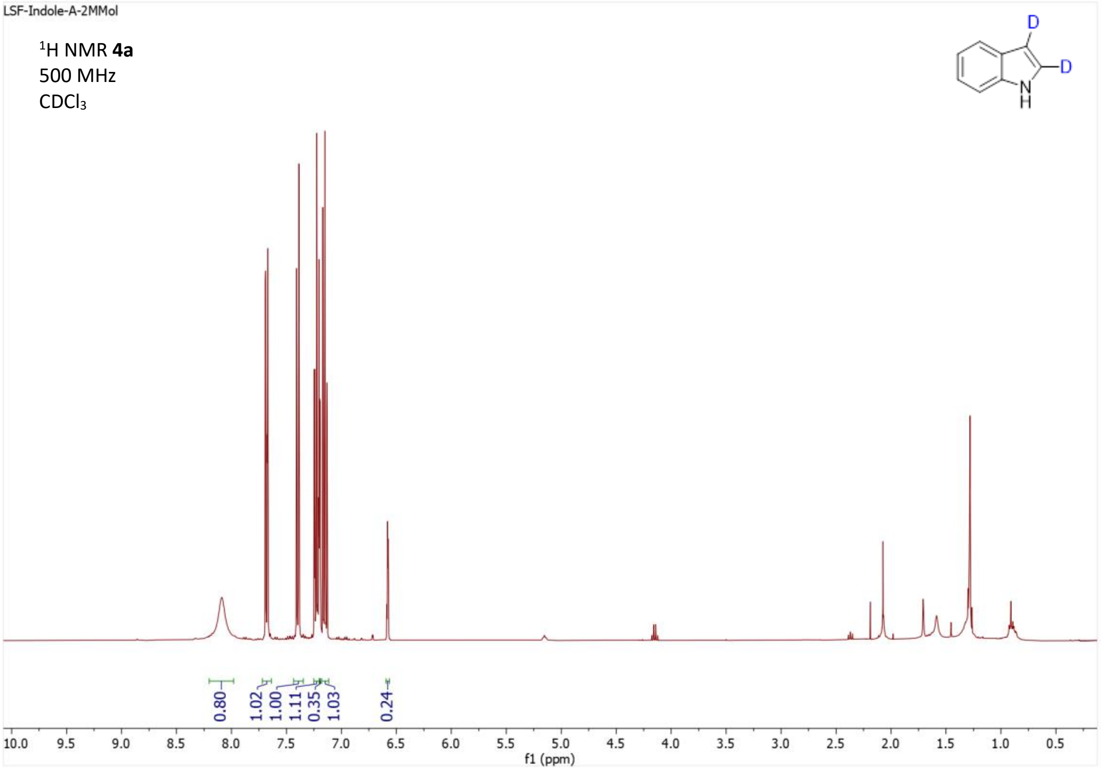

^{13}C NMR spectrum of (4a) (0.4 mmol scale)

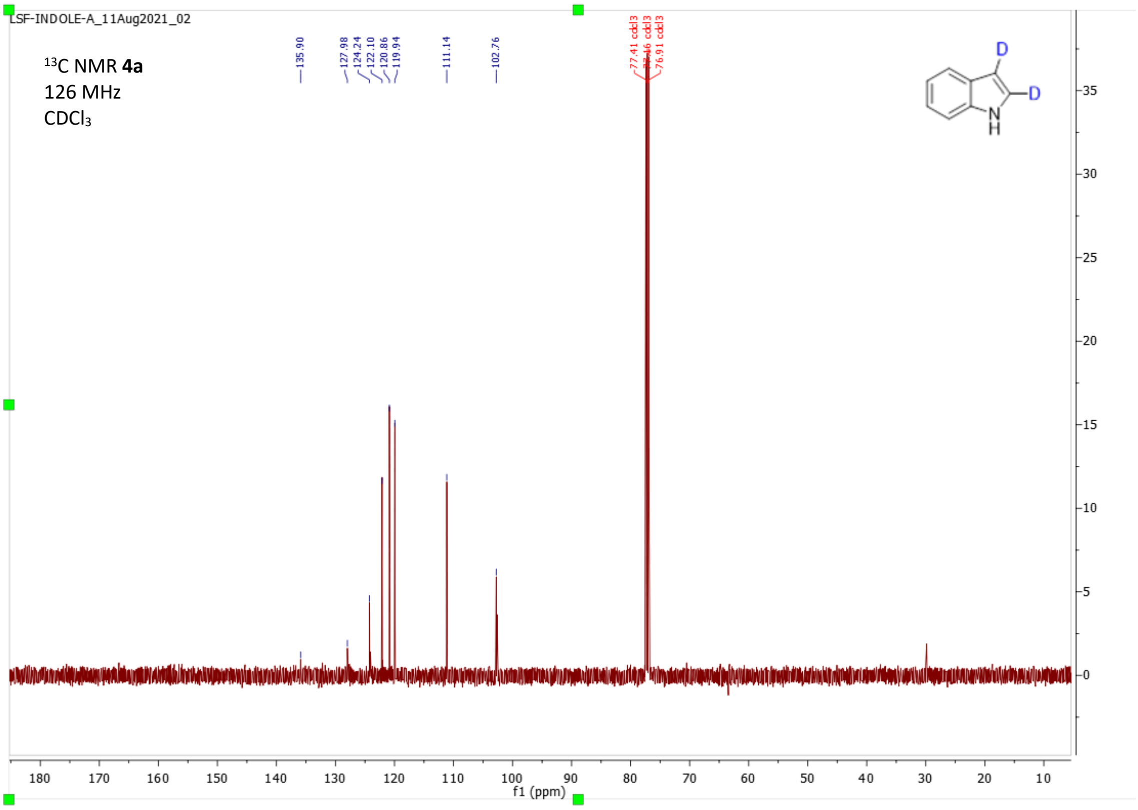

^{1}H NMR spectrum of (4c)

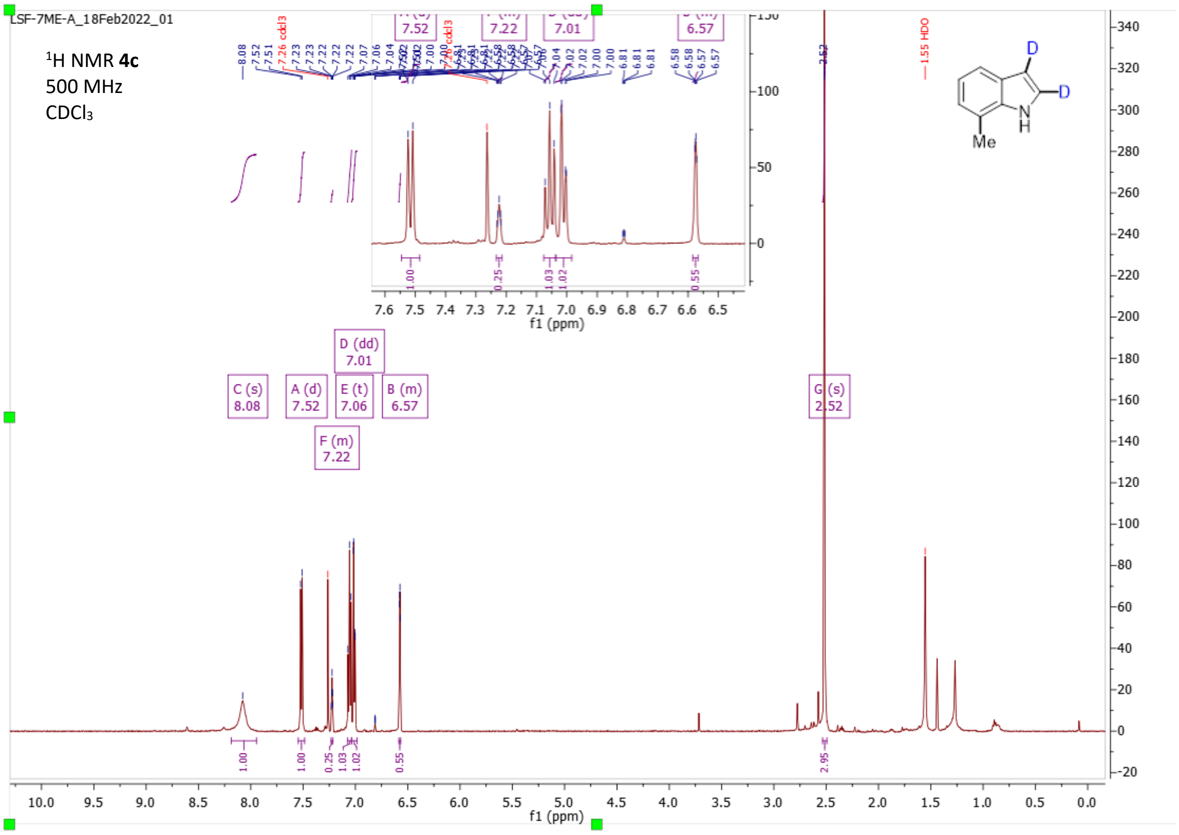

^{1}H NMR spectrum of (4d)

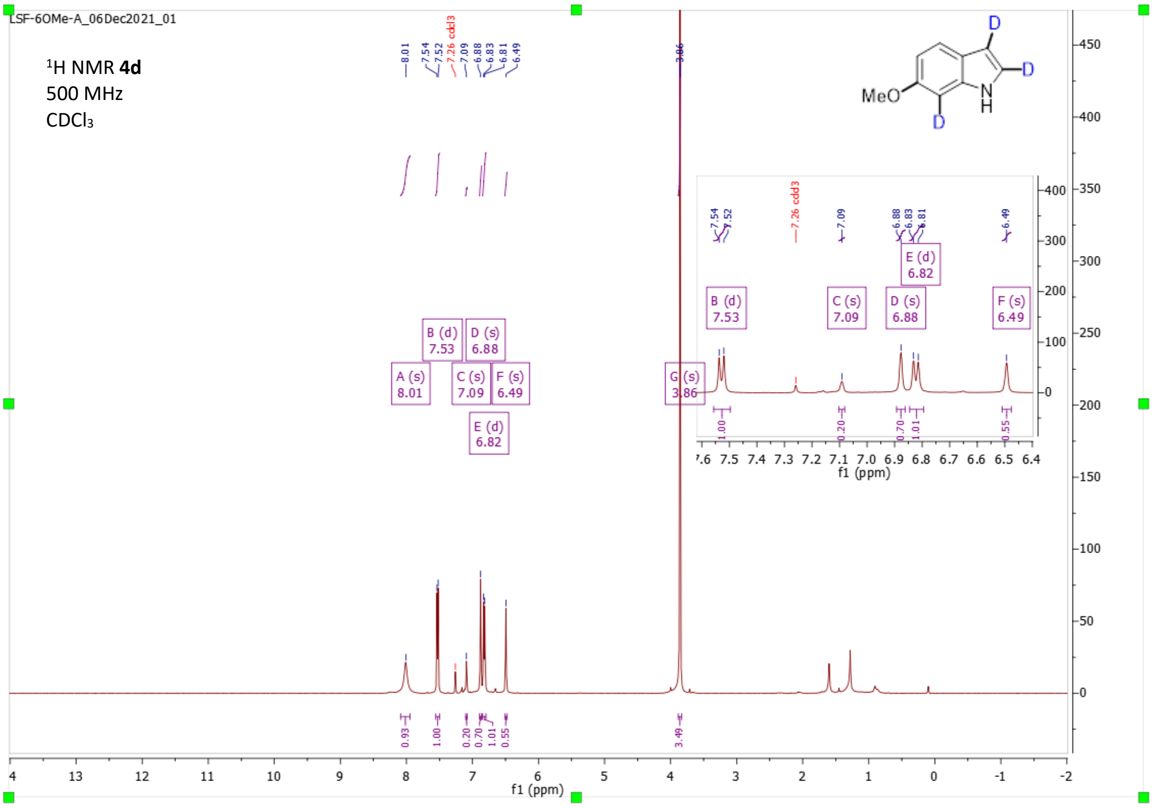

^{13}C NMR spectrum of (4d)

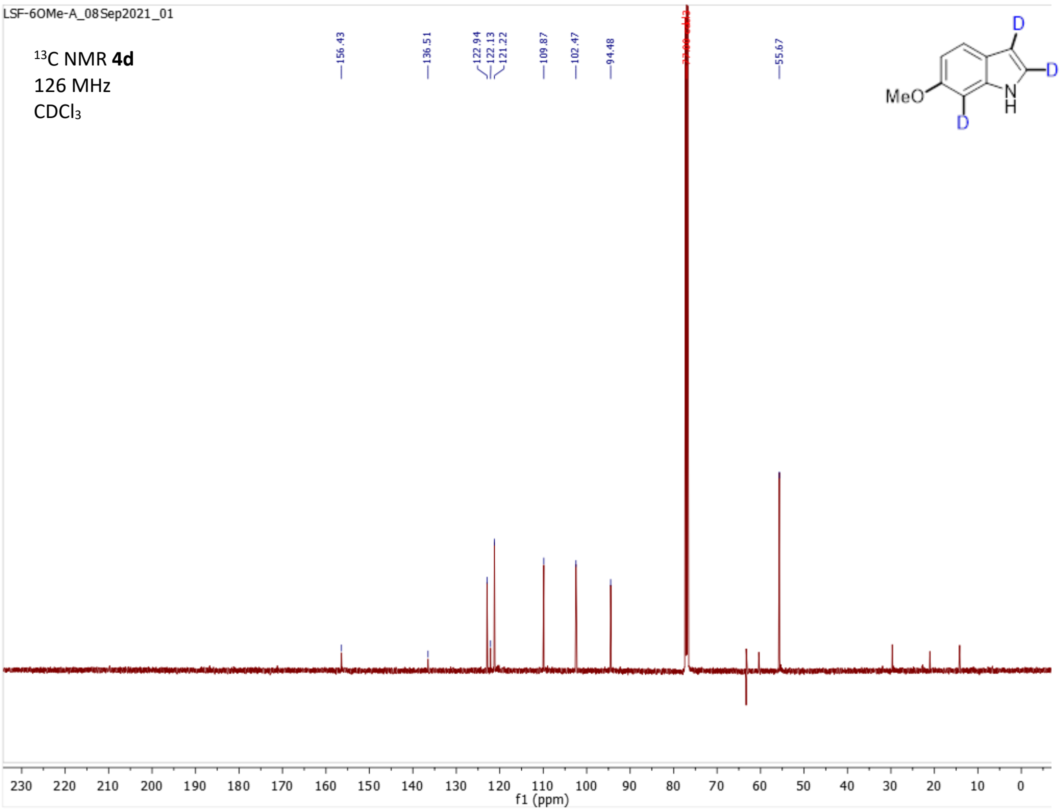

^{1}H NMR spectrum of (4e)

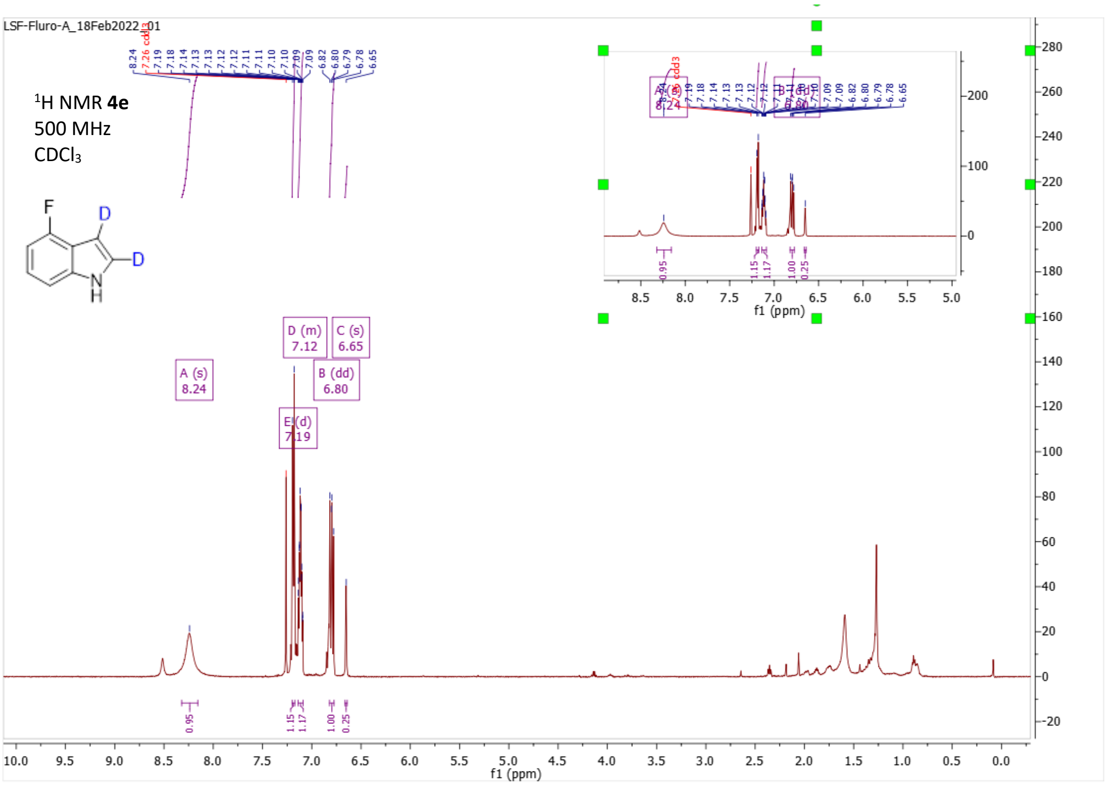

^{13}C NMR spectrum of (4e)

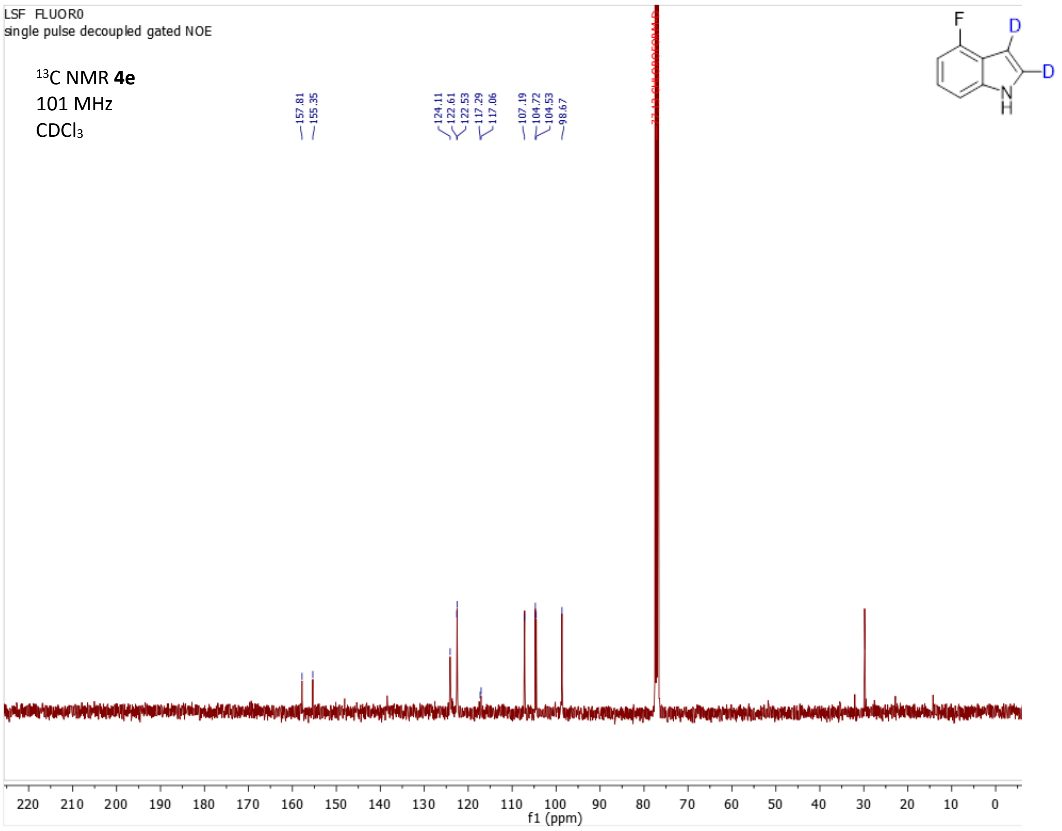

^{19}F NMR spectrum of (4e)

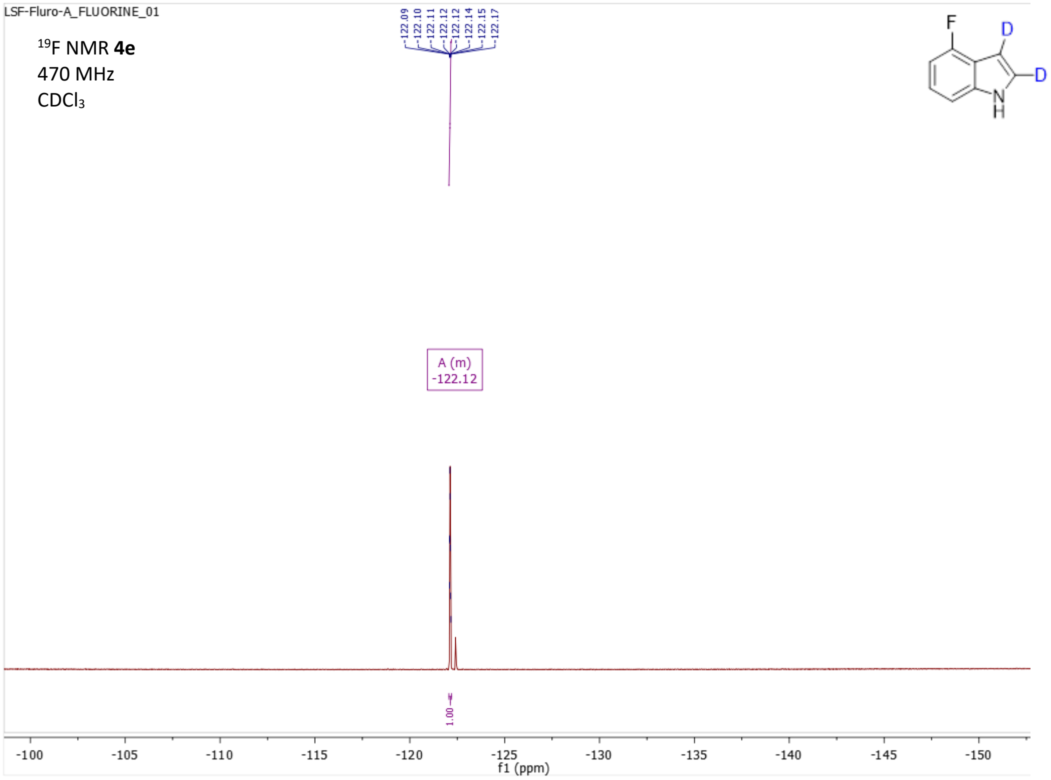

^{1}H NMR spectrum of (4f)

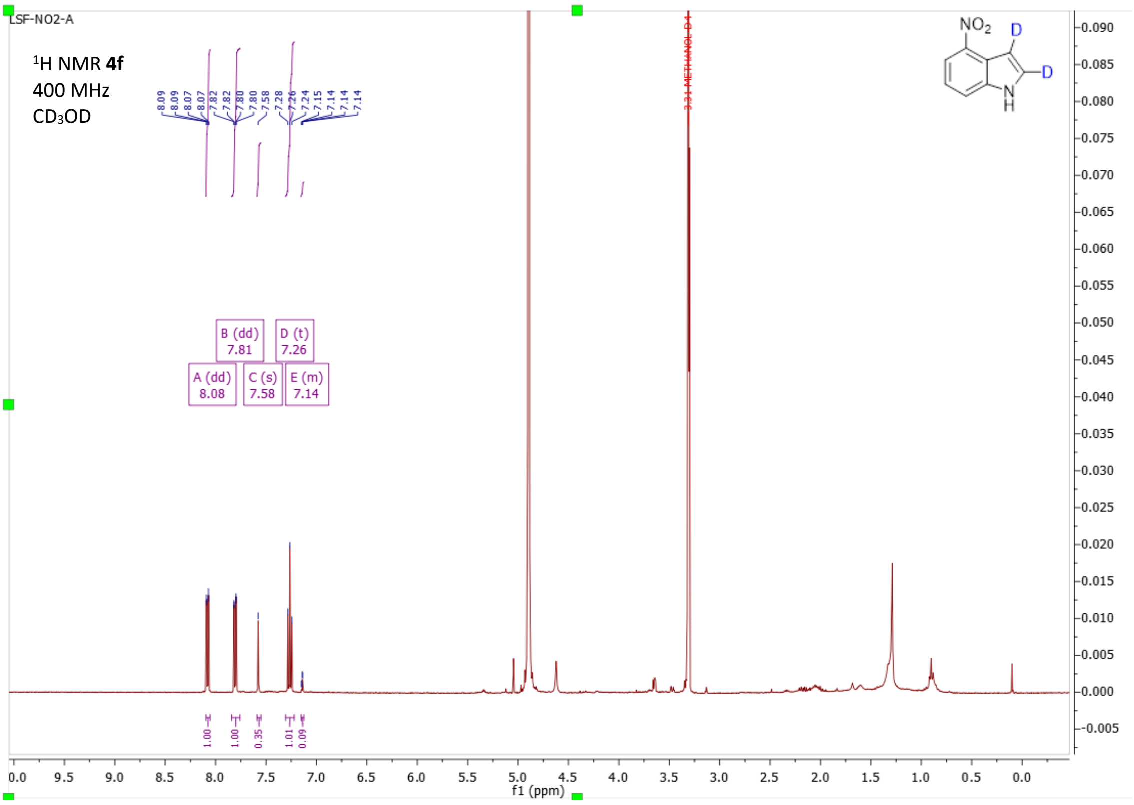

^{13}C NMR spectrum of (4f)

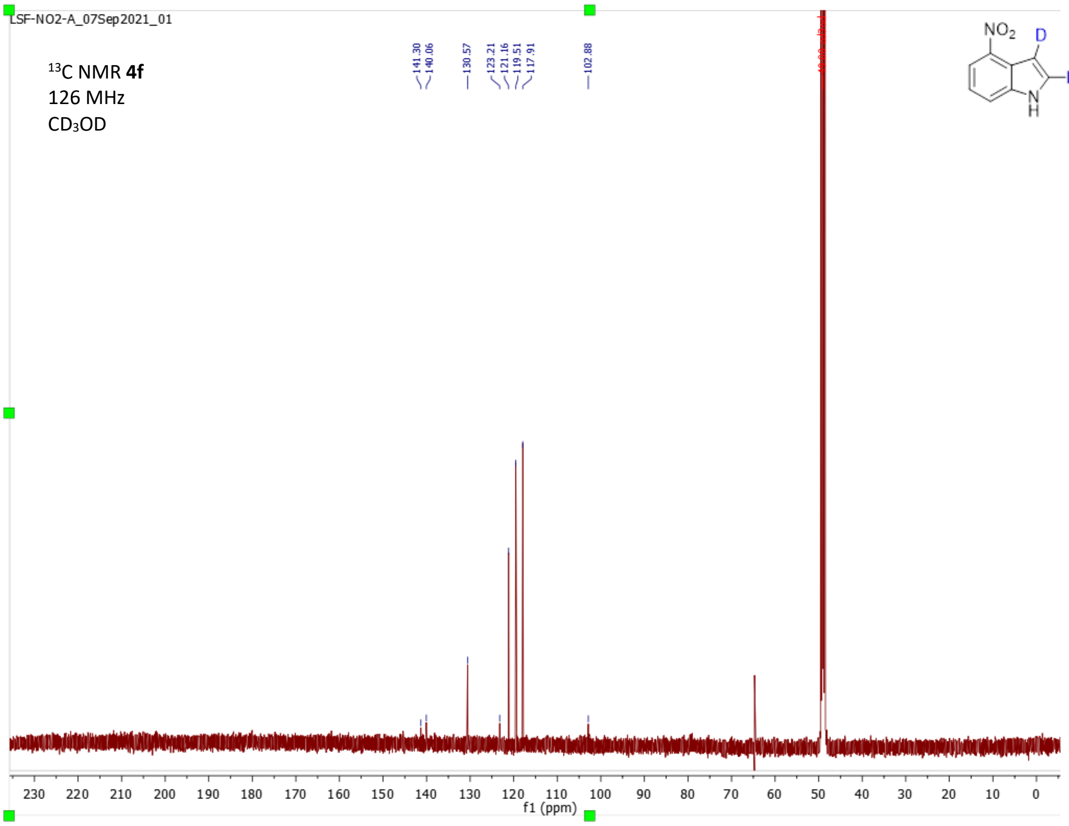

^{1}H NMR spectrum of (4i)

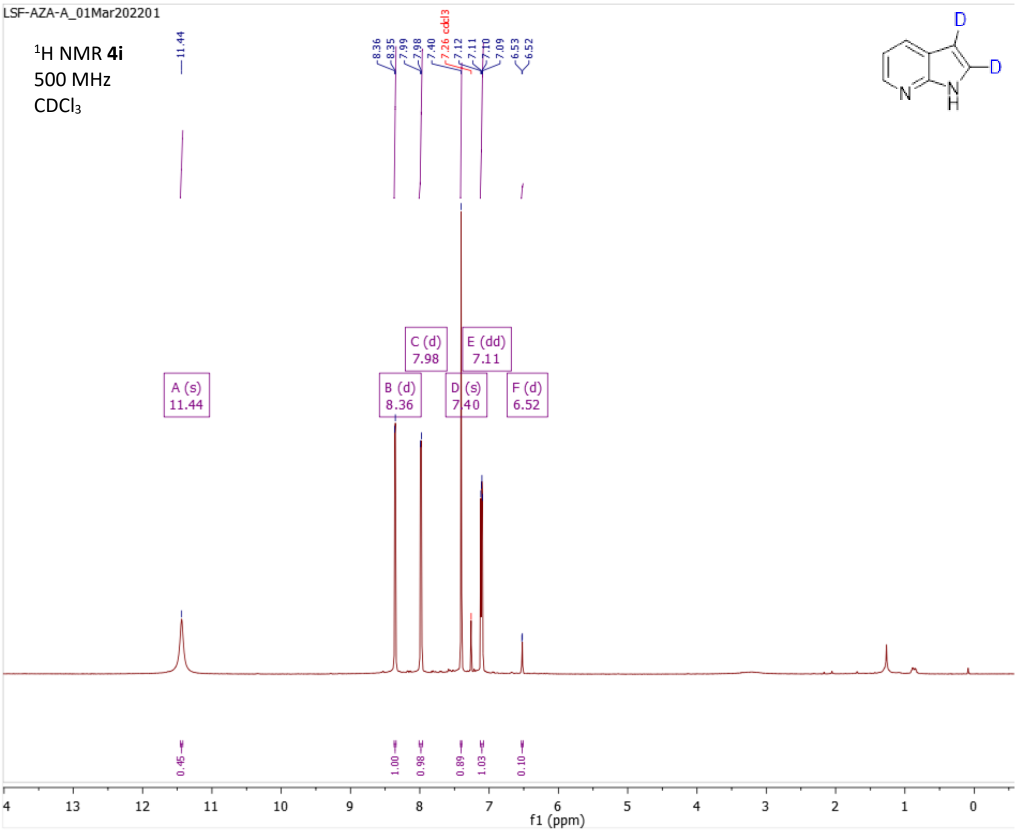

^{13}C NMR spectrum of (4i)

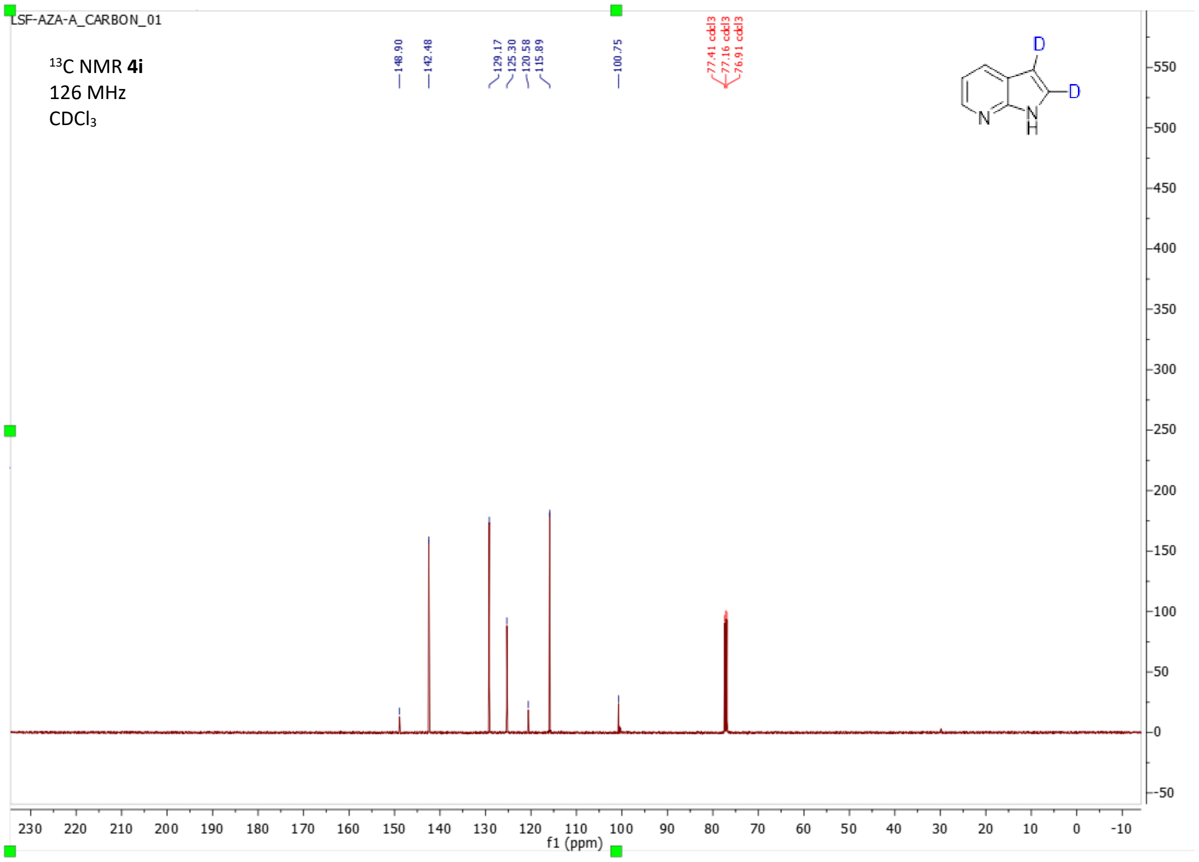

^{1}H NMR spectrum of (4l)

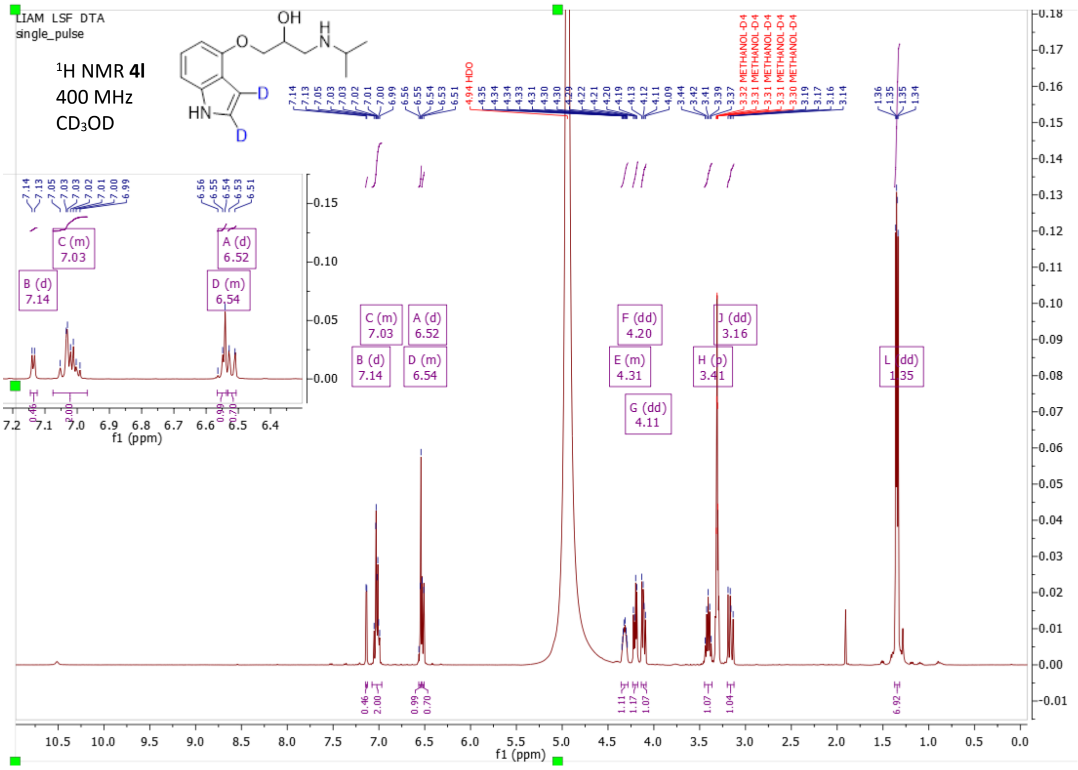

^{13}C NMR spectrum of (4l)

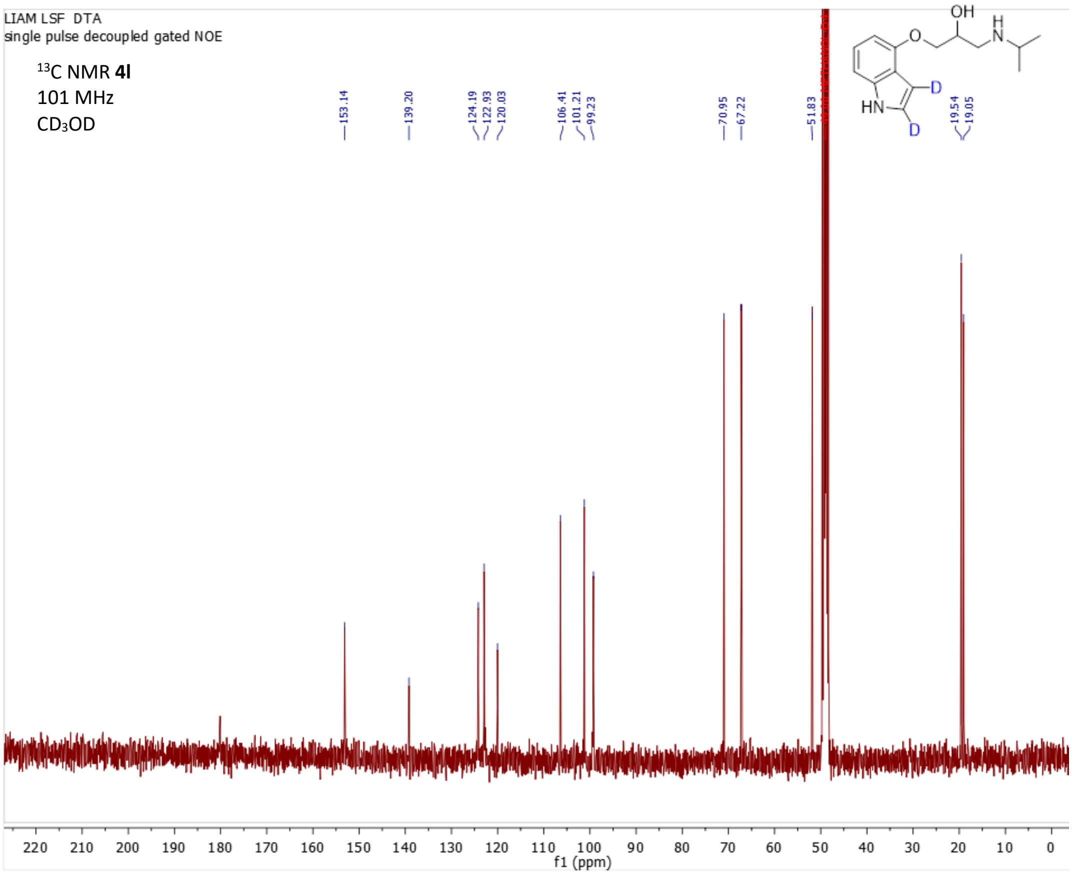

C3-Deuterated Indoles

^{1}H NMR spectrum of (2a)

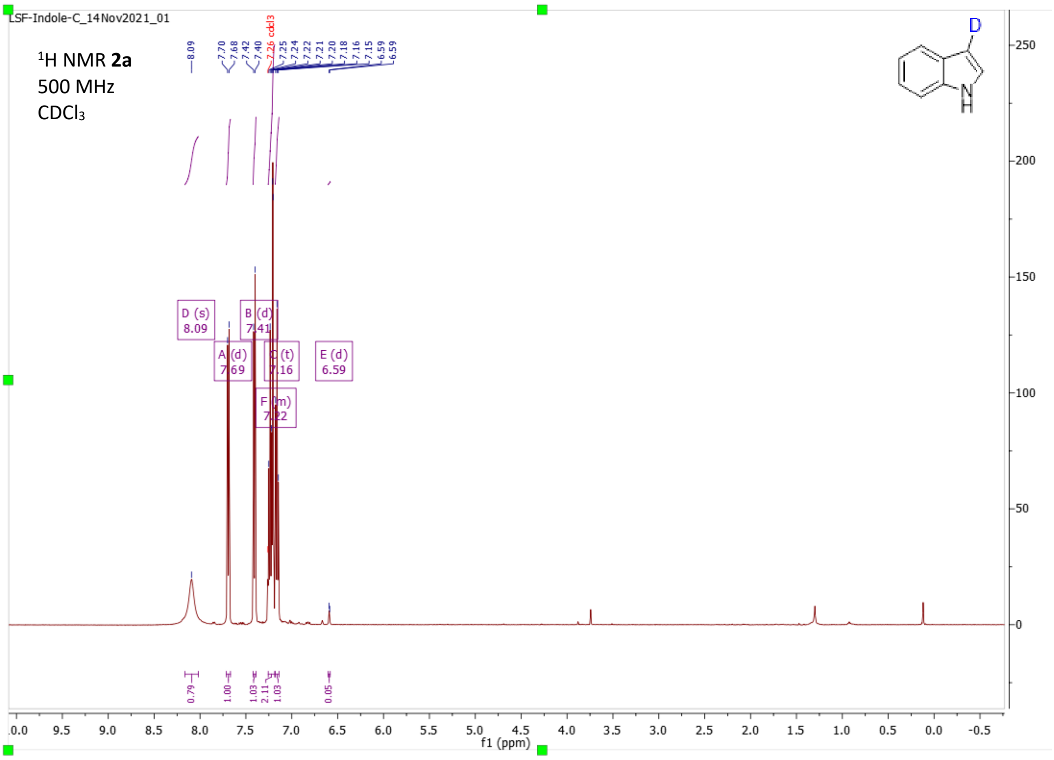

^{1}H NMR spectrum of (2c)

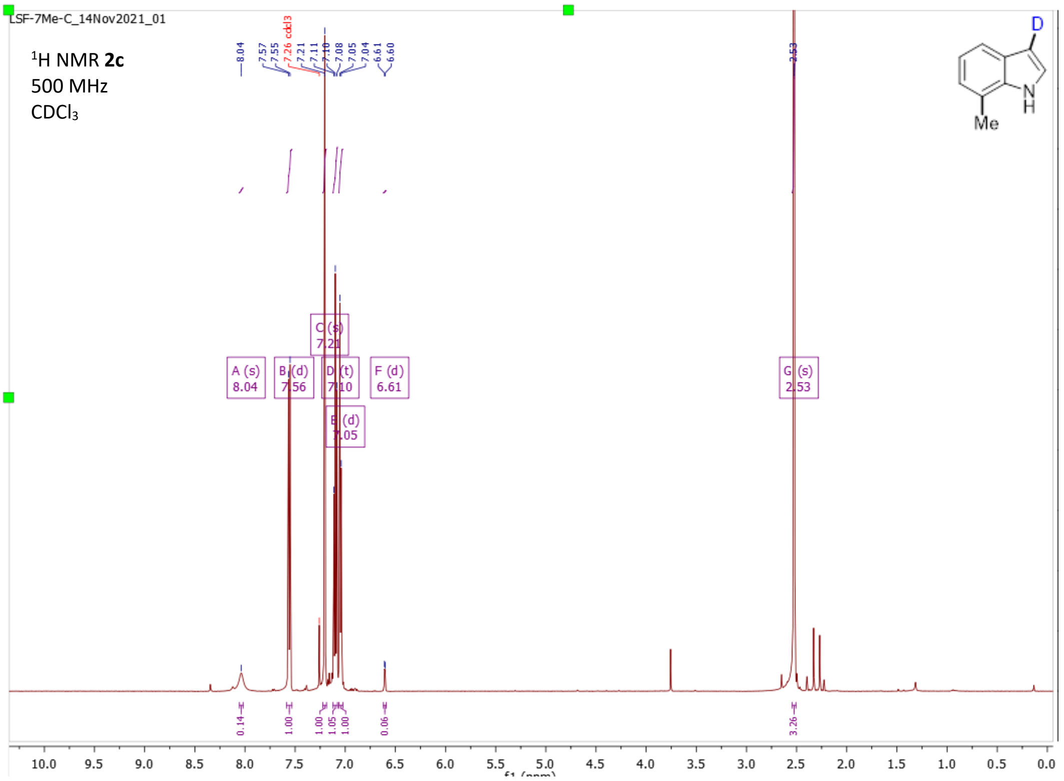

^{13}C NMR spectrum of (2c)

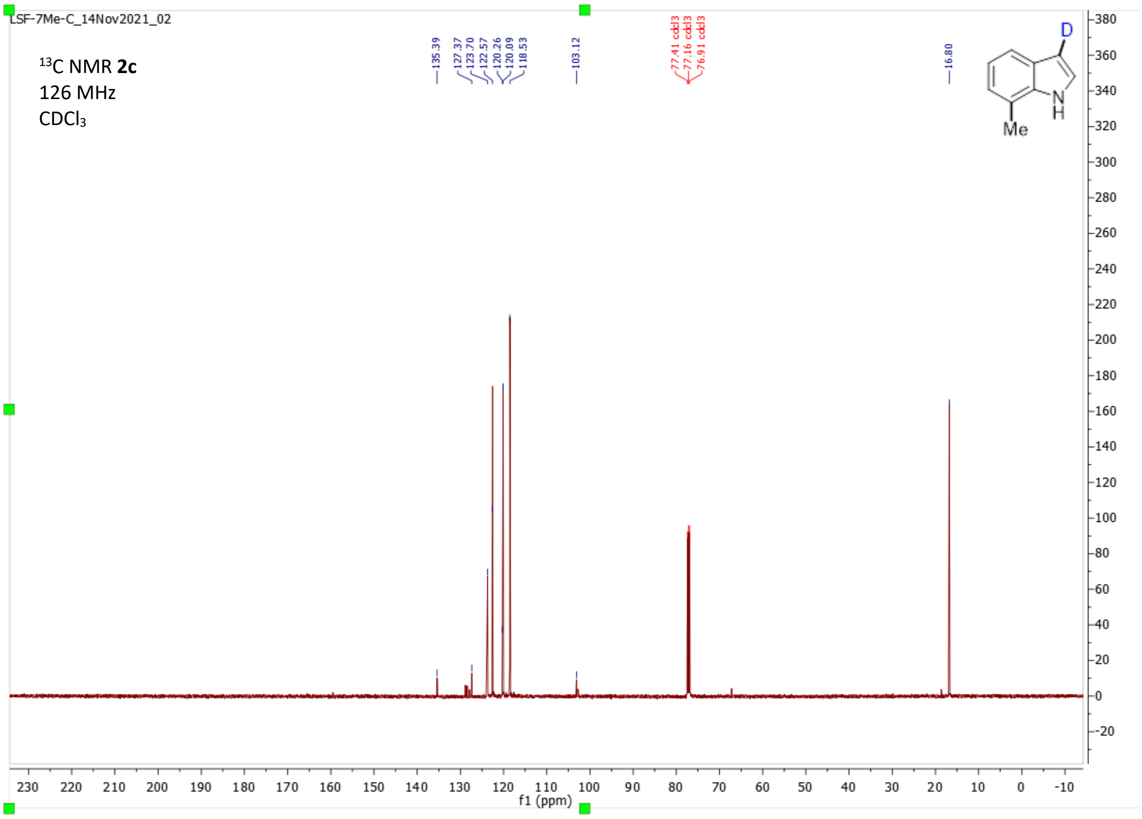

^{1}H NMR spectrum of (2d)

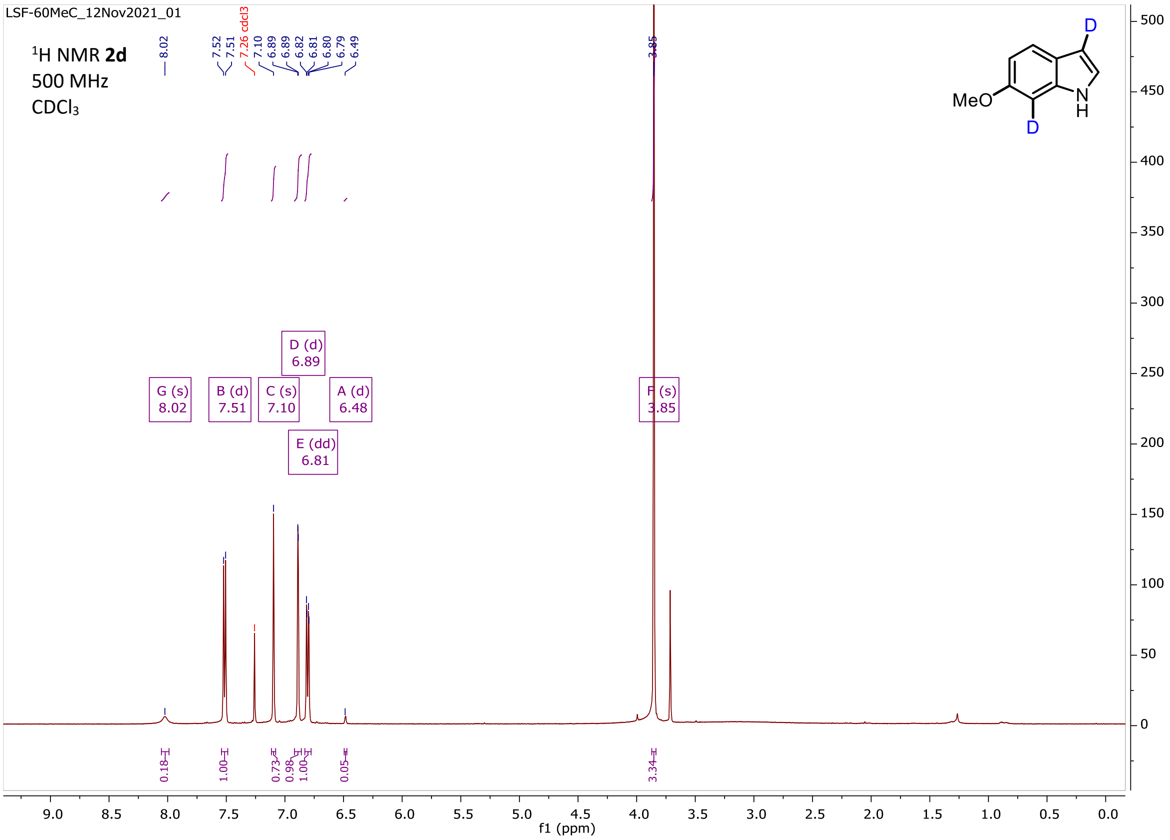

^{13}C NMR spectrum of (2d)

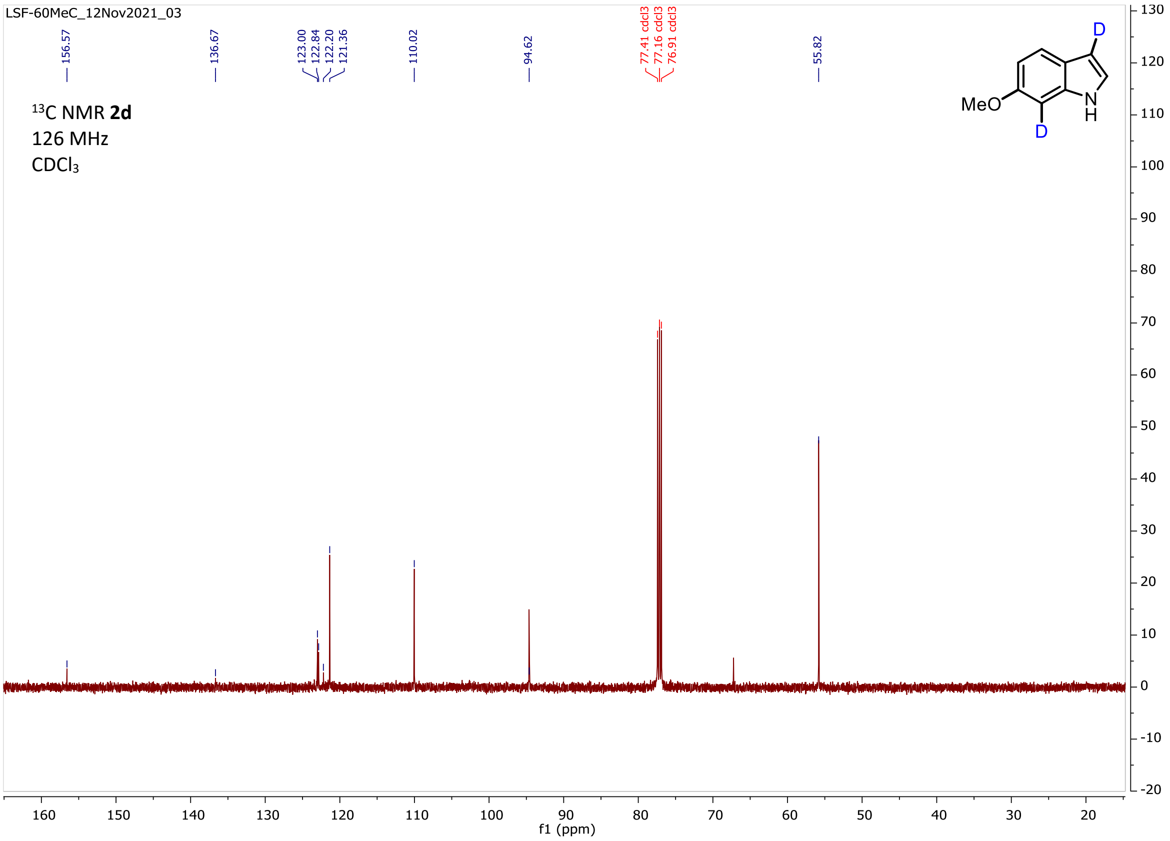

^{1}H NMR spectrum of (2e)

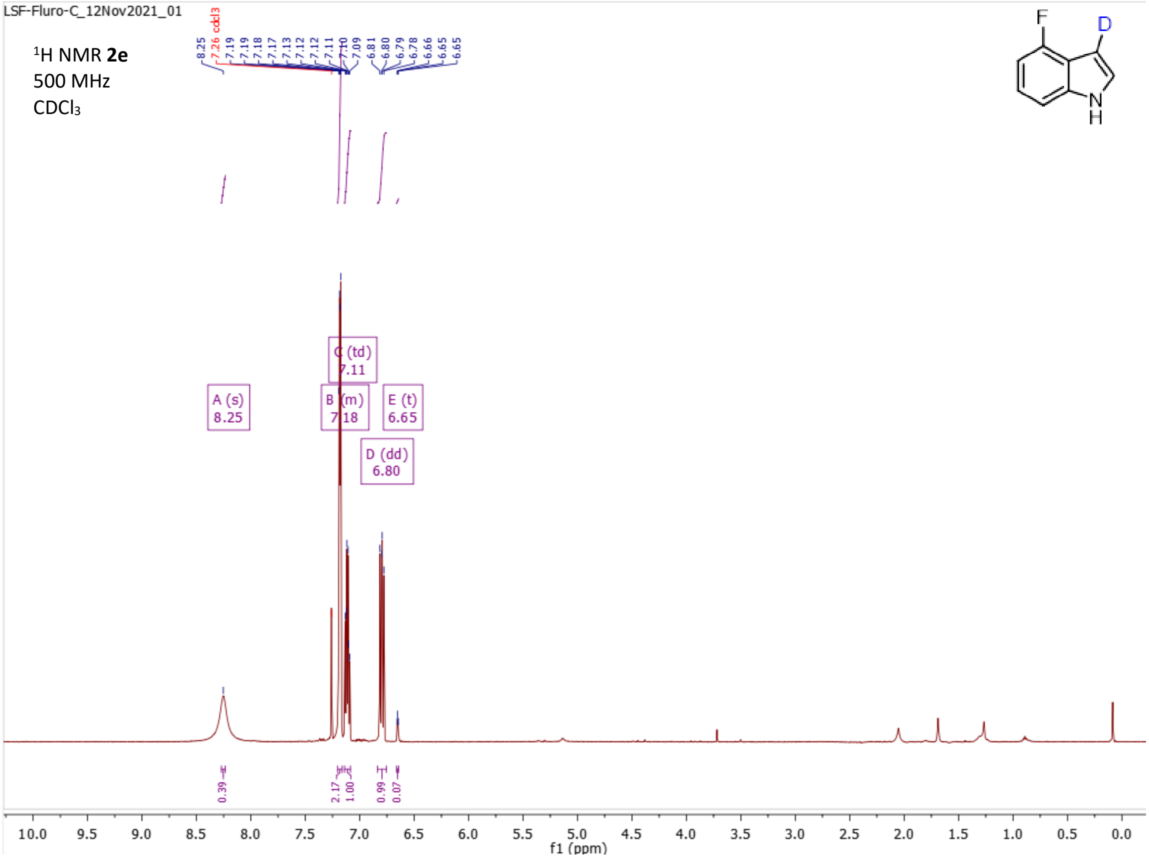

^{13}C NMR spectrum of (2e)

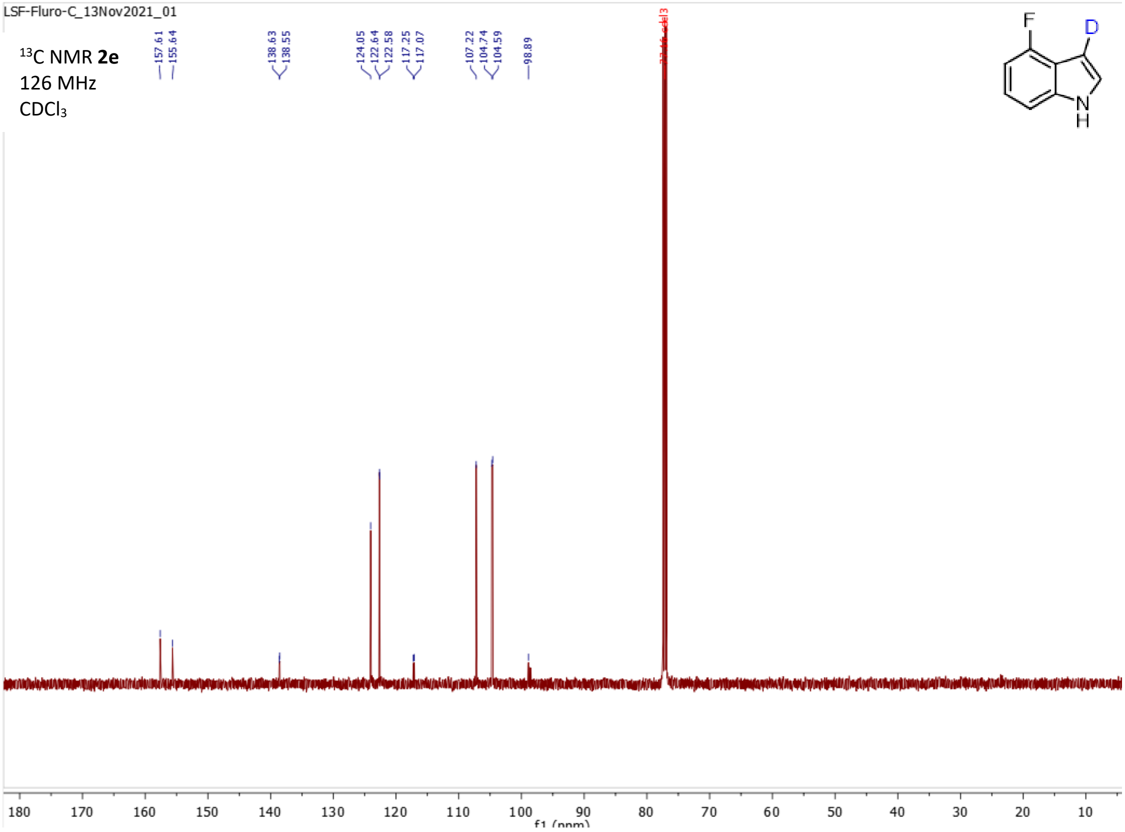

^{19}F NMR spectrum of (2e)

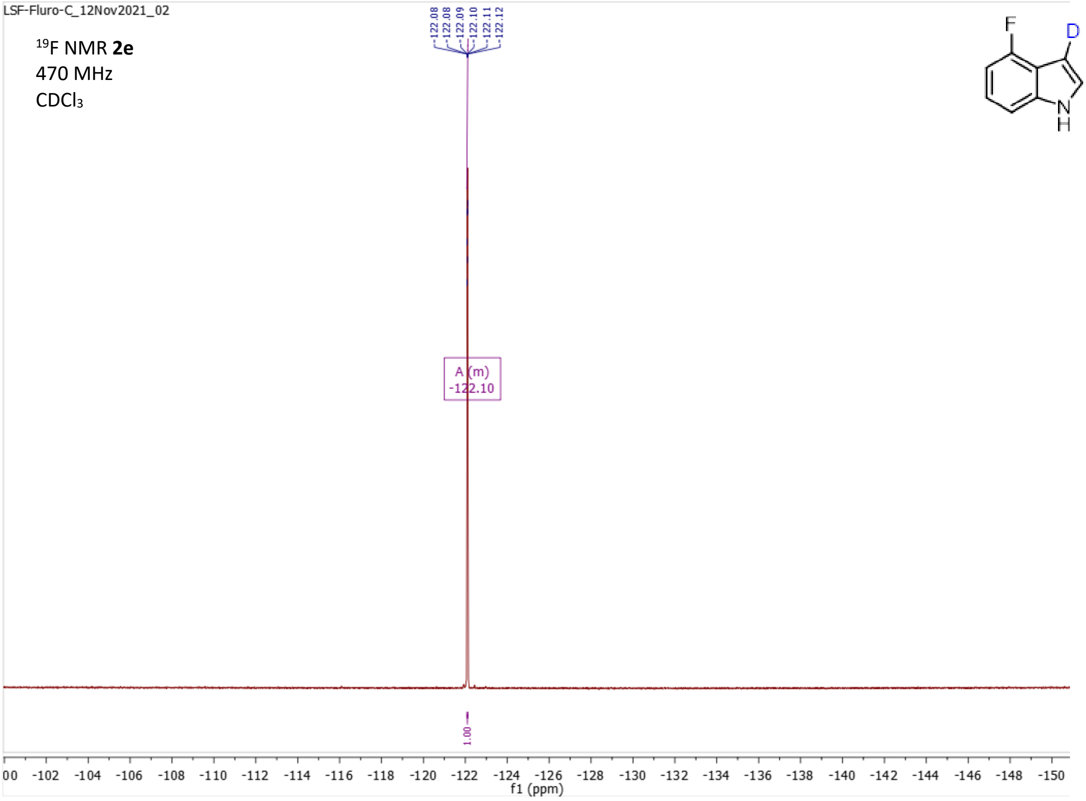

^{1}H NMR spectrum of (2f)

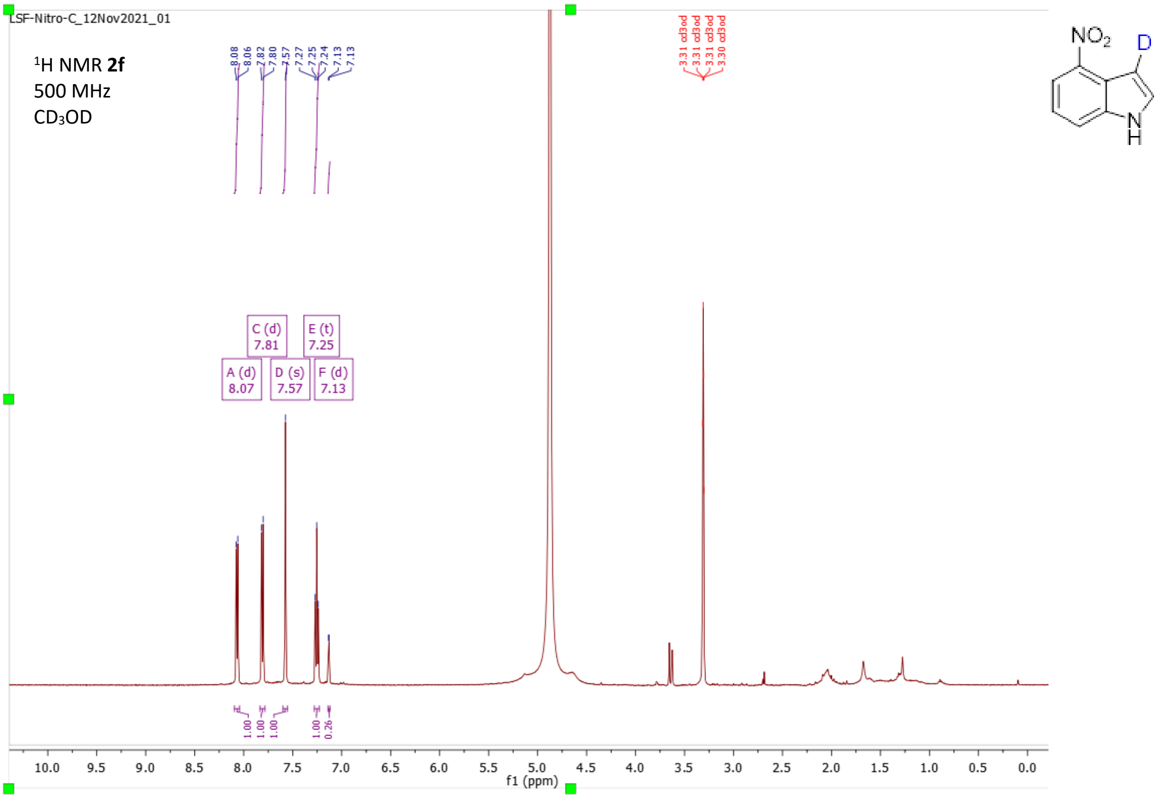

^{13}C NMR spectrum of (2f)

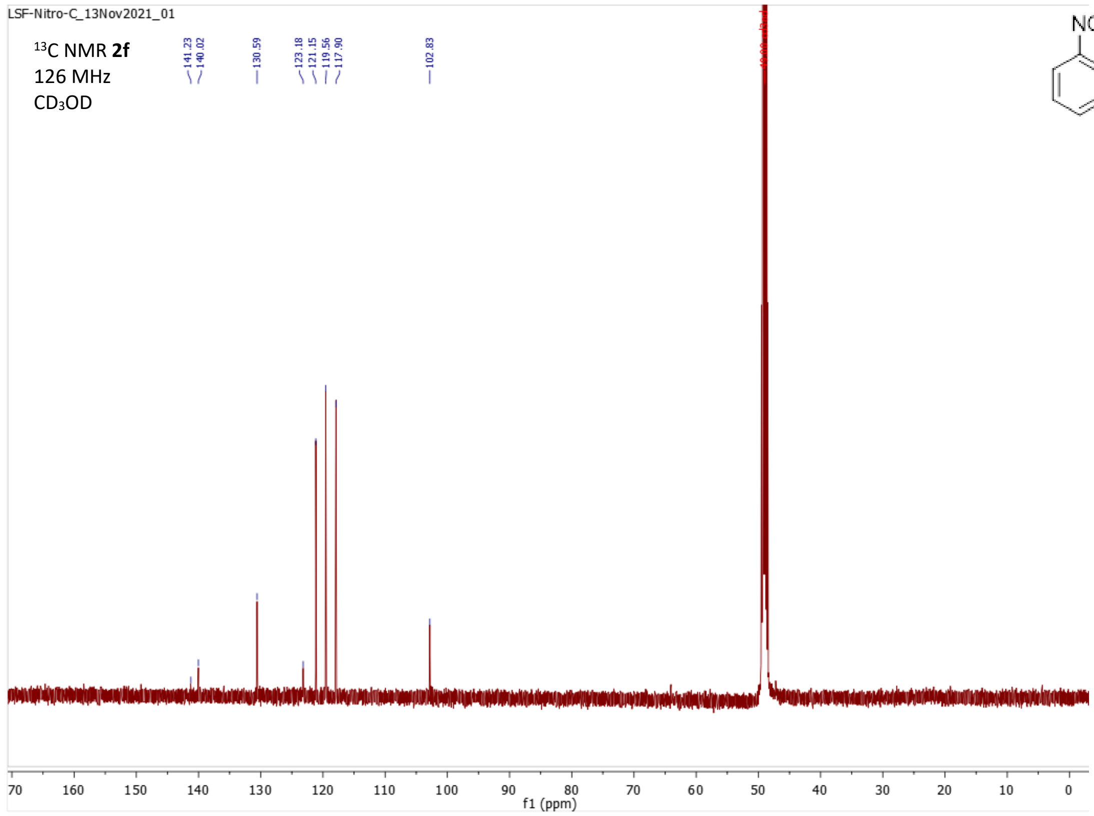

^{1}H NMR spectrum of (2i)

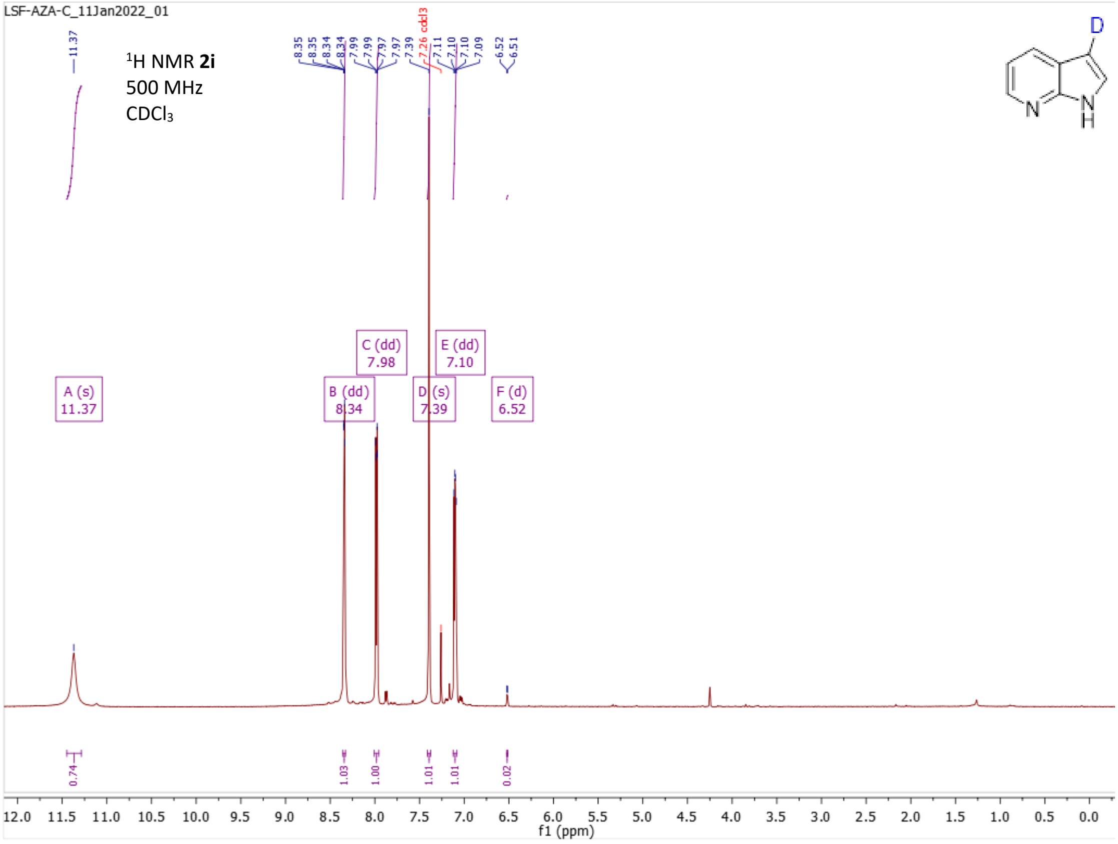

^{1}H NMR spectrum of (2l)

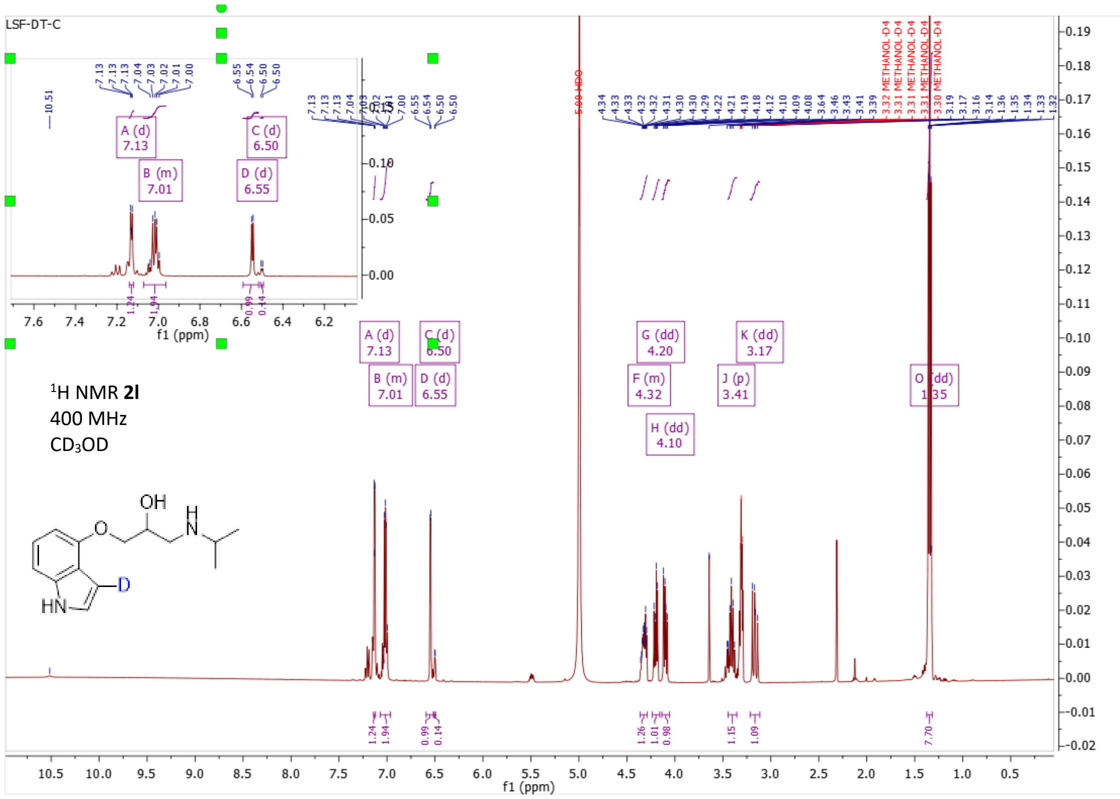

^{13}C NMR spectrum of (2l)

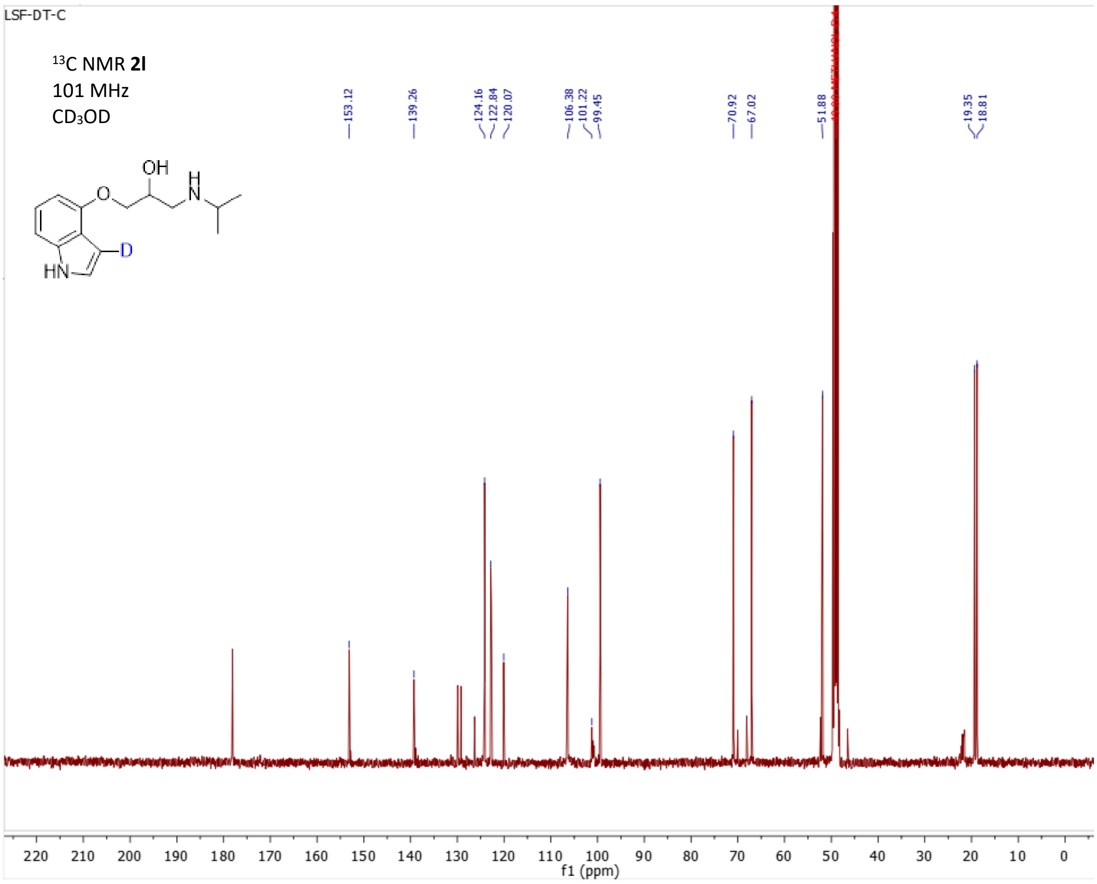

Supplement: Supplementary file 1 — jo3c00819_si_001.pdf [file jo3c00819_si_001.pdf]
